# Gene Drive for Mosquito Control: Where Did It Come from and Where Are We Headed?

**DOI:** 10.3390/ijerph14091006

**Published:** 2017-09-02

**Authors:** Vanessa M. Macias, Johanna R. Ohm, Jason L. Rasgon

**Affiliations:** 1 Department of Entomology, Pennsylvania State University, University Park, PA 16802, USA; jlr54@psu.edu; 2 Center for Infectious Disease Dynamics, Pennsylvania State University, University Park, PA 16802, USA; jo.ohm@psu.edu; 3 The Huck Institutes of the Life Sciences, Pennsylvania State University, University Park, PA 16802, USA

**Keywords:** CRISPR/Cas9, gene editing, vector control

## Abstract

Mosquito-borne pathogens place an enormous burden on human health. The existing toolkit is insufficient to support ongoing vector-control efforts towards meeting disease elimination and eradication goals. The perspective that genetic approaches can potentially add a significant set of tools toward mosquito control is not new, but the recent improvements in site-specific gene editing with CRISPR/Cas9 systems have enhanced our ability to both study mosquito biology using reverse genetics and produce genetics-based tools. Cas9-mediated gene-editing is an efficient and adaptable platform for gene drive strategies, which have advantages over innundative release strategies for introgressing desirable suppression and pathogen-blocking genotypes into wild mosquito populations; until recently, an effective gene drive has been largely out of reach. Many considerations will inform the effective use of new genetic tools, including gene drives. Here we review the lengthy history of genetic advances in mosquito biology and discuss both the impact of efficient site-specific gene editing on vector biology and the resulting potential to deploy new genetic tools for the abatement of mosquito-borne disease.

## 1. Toward Gene drive for Vector-Borne Disease Control: A History

### 1.1. Initial Proposals for Genetic Control of Insect Vectors of Disease

Vector control is critical to the reduction vector-borne diseases [1,2]. When mosquitoes were identified as vectors of the pathogens causing diseases such as malaria and yellow fever, efforts were undertaken to eliminate mosquitoes from disease-endemic regions [3,4,5,6]. Prior to the 1940s, tools to target mosquitoes for elimination were limited to environmental manipulations and non-persistent insecticides. With these tools, successful efforts were made to eliminate both *Anopheles* and *Aedes* species from Cuba, Panama, Brazil and later nearly the entirety of the Americas. [7,8,9]. In 1939 the discovery of DDT (dichloro-diphenyl-trichlorethane) as a persistent insecticide for use against mosquitoes introduced the possibility of sustainable mosquito control and a new objective emerged: global malaria eradication. With this outlook came the impetus to embark on the first global malaria eradication campaign in the 1950s which achieved a remarkable re-drawing of the malaria incidence lines. However, within a decade of its deployment, DDT resistance had emerged alongside social concerns that insecticides caused harm to the environment and human health [10,11].

The incredible successes in regional elimination and the ultimate failure to achieve global malaria eradication, provided the context for an interest in genetic approaches to mosquito control. Proposed genetic methods for pest population suppression were inspired by natural genetic phenomena, a trend that continues in the development of molecular tools today. The earliest recognition was made by Serebrovskii, while working with Muller, who realized that X-ray-induced chromosomal translocations caused sterility in offspring heterozygous for the translocation [12,13] and by Knipling who, while working for the USDA on the screwworm, *Cochliomya hominvorax*, observed that the agricultural pest was monogamous and males of the species were sexually aggressive [14]. These observations along with Muller’s insect X-ray sterilization procedures, led to a screwworm sterilization program that was the basis of the Sterile Insect Technique (SIT), an approach to pest control that eventually led to the elimination of the screwworm from the entire continent of North America in 1968 [14,15]. Concurrently, Vanderplank began work on reducing tsetse fly populations when he recognized that two species (*Glossina morsitans* and *G. swynnertoni*) from different regions could mate, but that the offspring were partly sterile. Releases of each species into the other’s regions led to local elimination in both regions [16,17,18].

### 1.2. Early Definitions and Perspectives on Genetics for Vector Control

In 1964, the WHO defined genetic control as “the use of any condition or treatment that can reduce the reproductive potential of noxious forms [of the insect] by altering or replacing the hereditary material.” This definition encompasses both categories of genetics-based strategies into which today’s approaches fall: *population suppression*, which includes strategies to reduce or eliminate an insect population, and *population modification*, which involves replacing existing wild mosquito populations with strains or species that are innocuous in terms of pathogen transmission (also called “replacement” or “alteration”) [19]. Both population suppression and modification in this era were imagined to be manipulations of existing phenotypes and genetic phenomena [20,21]. Early proponents of population suppression by genetic means envisioned releasing mosquitoes that would produce sterile offspring after mating with wild mosquitoes, either by cytoplasmic incompatibility or by hybrid sterility as in Vanderplank’s tsetse fly experiments. Roles were proposed for genetic elements with interesting inheritance phenotypes, such as sex-ratio distorters that could male-bias a population into extinction. Meiotic drive, a sex-linked genetic component identified in mosquitoes in 1967, showed biased inheritance patterns and was imagined as a mechanism to drive detrimental genes into insect populations [22,23].

Enthusiasm was not as high for population modification strategies. Examples of species being replaced by invasion of other insect species gained the strategy special mention in the WHO’s 1964 report, but these weren’t characterized thoroughly enough to inspire confidence in intentionally affecting the replacement of vector mosquitoes with innocuous strains or species. However, Wood et al. showed in 1977 that a gene could be driven into an *Aedes aegypti* cage population using negative heterosis/underdominance, a phenomenon whereby reduced survival of heterozygotes allowed a skewing of the population toward released mosquitoes [24,25,26]. These demonstrations along with investigations into the biological basis of mosquito refractoriness to vector-transmitted pathogens stimulated renewed enthusiasm for population modification strategies [23,27,28].

### 1.3. The Birth (or Coming-of-Age) of a New Field: Vector Biology Leverages Natural Genetic Phenomena to Develop Important Tools in Mosquito Genetics

A range of possibilities for genetics-based vector control was documented in these early studies, but much had yet to be done. The field of vector-borne disease research was not highly populated or well-funded. By the 1980s vector-borne diseases were re-emerging into previously controlled or non-endemic areas and there was still a lack of vaccines and therapeutics for many of these diseases. Studies in vector biology and the development of genetics-based tools for vector control were seen as a mostly untapped, but critical potential source of new disease-control strategies. In response to the stagnancy in vector work, a group of scientists proposed to stimulate the field by integrating researchers with relevant, but disparate expertise into vector biology and by moving technology that had been introduced in other genetic systems into vector biology. Thus the Vector Biology Network (VBN) was formed, the activities of whom led to a remarkable growth in genetic technologies for mosquitoes and primed the field for the application of modern genetic manipulation to the problem of vector-borne diseases [29]. Following the establishment of the VBN, a boom in funded research and scientific reports reflected the rapidly growing knowledge in vector molecular biology and genetics [29].

The introduction of new advances in biotechnology to the field of vector biology was predicted to lead to new approaches to vector control. Indeed, an entire genre of synthetic mechanisms, including a vision for gene drive systems were developed, inspired by the discovery and characterization of naturally-occurring selfish genetic elements [30,31,32,33]. These that are inherited more frequently than expected by Mendelian inheritance patterns and so have the potential to overcome evolutionary constraints and spread desired genotypes into vector populations either for suppression or modification strategies. The last half of the 20th century would see many developments based on selfish genetic elements [34].

#### 1.3.1. Transposons as a Basis for Genetic Transformation

In the 1950s, McClintock’s work revealed that genes called transposons could mobilize within a genome [35]. We now understand that some transposons can spread quite rapidly through populations despite severe costs to the host [36,37,38,39]. This finding was key to insect transformation, which was initially achieved using the *P*-element in *Drosophila melanogaster* [40]. Non-drosophilid insects were more difficult to transform as the *P*-element wasn’t active in other insects, few visible markers to screen for transformants were available, and many insect-rearing requirements were less straight-forward than for drosophilids [41]. By 2002, transformation of several mosquito species was achieved using transposons identified as active in mosquitoes: *Hermes* elements in *Aedes aegypti* (1998) and *Culex quinquefasciatus* (2001), a mos1/mariner element in *Ae. aegypti* (1998), and the *piggyBac* element in *Anopheles stephensi* (1999), *An. gambiae* (2000), *Cx. quinquefasciatus (2001) An. albimanus* (2002), *Ae. fluviatilis* (2006) and *Ae. albopictus* (2010) [42,43,44,45,46,47,48,49,50]. New transformations in mosquitoes were complimented by continued description of useful promoters and gene control elements, the advancement of sequencing technology, and the application of fluorescent markers, without which most transposon insertions would be undetectable (for a comprehensive promoter list see Table 8.2 in [51]). It was imagined that transposons would also be useful as a gene drive system, but transposons that could mediate insertion into a mosquito’s genome were not so easily remobilized [52,53,54,55,56,57]. Only recently has a synthetic construct based on the *piggyBac* transposon been demonstrated to mobilize itself once inserted into a mosquito genome, but rarely [58,59].

#### 1.3.2. The Skewing of a Population toward Desirable Genotypes Using Underdominance and Cytoplasmic Incompatibility

Underdominance was described in early papers as negative heterosis, the phenomenon of heterozygotes being less fit than homozygotes; it was proposed that a target population could be modified if genetic components that caused negative heterosis were linked with a desirable trait and introduced at a high enough proportion. After much of the resulting population was diminished (all heterozygotes), the allele highest in abundance would increase to fixation [20]. Curtis explored this in the lab and in the field using translocations [24,28]. A synthetic design where underdominance is encoded as two separate gene constructs, both linked to the effector gene to be driven, was proposed by Davis et al. in 2001 and successfully developed into a *Drosophila* gene drive strain by Akbari et al. in 2013, but has not yet been demonstrated in mosquitoes [60,61]. Cytoplasmic incompatibility can similarly drive the fixation of a genotype of a released strain by reducing the survival of offspring from parents with differing *Wolbachia* infection status [62,63,64], and was proposed to be recapitulated using *Wolbachia* genes in transformed mosquitoes, but these have only recently been identified using comparative genomics of *Wolbachia* strains in *Drosophila* [65,66,67]. Transformation of *D. melanogaster* with these genes produced cytoplasmic incompatibility phenotypes; it is possible that this strategy will be feasible in mosquitoes in the near future [65].

#### 1.3.3. The Application of Naturally Occurring Selfish Elements to Synthetic Gene drives in Mosquitoes

Naturally occurring selfish elements called Maternal Effect Dominant Embryonic Arrest (MEDEA) were discovered to spread through populations of the flour beetle, *Tribolium castaneum,* by segregation distortion, favoring offspring that encode the element [68,69]. In this insect, a single MEDEA locus was described to bear genes encoding both a maternal product that is lethal to developing embryos and a zygotic product that rescues offspring from the lethal product, so that only progeny bearing the locus would survive to adulthood; this system inspired engineering of the first successful demonstration of a synthetic gene drive [70].

Mobile introns were described in the 1970s in yeast that were capable of “homing”; they could be copied laterally to a homologous sequence and so increase their presence in offspring [71,72,73]. Site-specificity was attributed to endonuclease recognition of DNA sequence [74]. These elements called homing endonuclease genes (HEGs) are widespread and demonstrate the non-Mendelian inheritance that is attractive for vector control applications. In 2003, HEGs were proposed as a useful basis for gene drive [75] and work was already underway to modify the endonucleases to recognize specific insect sequences [76,77,78]. Successful engineering of a HEG-based gene drive in *An. gambiae* was reported in 2011 [79].

#### 1.3.4. The Application of the Endosymbiont *Wolbachia* to Gene drive in Mosquitoes

The bacterial endosymbiont *Wolbachia pipentis* was identified as the cause of reproductive phenotypes described as early as 1967 that bias inheritance of the bacteria in crosses between infected and uninfected mosquitoes [62,63,64,80]. The bacteria’s reproductive phenotypes can lead to their spread into an insect population. *Wolbachia* has thus been proposed as a driver for a synthetic gene construct, but thus far the bacteria have not been amenable to transformation [67,81,82,83]. However, the reproductive phenotypes of the endosymbiont have provided useful strategies to combat mosquito-borne disease, both for mosquito population suppression and modification. The *w*Pip strain of *Wolbachia* isolated from *Cx. pipiens Linnaeus* has been used to infect *Ae. albopictus* and infected males are being used in a strategy analogous to SIT. The strategy is based on the observation that *w*Pip infected males mate with and sterilize wild females, which are naturally infected with other strains of *Wolbachia* [80,84,85,86,87,88]. *Wolbachia*’s drive potential paired with the discovery that the *Wolbachia* strain *w*Mel can block the development of dengue in *Ae. aegypti* and thereby interrupt transmission of the virus has led to a population modification strategy based on a combination of pathogen-blocking and gene drive. Releases of *Wolbachia*-infected mosquitoes in Australia, Vietnam, Colombia and Brazil have been some of the most widespread and effective examples of population modification in wild vector populations [89,90,91,92].

The *Wolbachia*-based programs have, in some ways, benefited from drive elements already crafted in nature to be effective. Still, a good amount of human ingenuity goes into discovery, description and assessment of the mosquito phenotypes and drive characteristics, and some engineering can be required for successful application. An additional advantage of these platforms is that release of mosquitoes carrying *Wolbachia* is perceived as a biocontrol agent and regulated as a microbial biopesticide, similar to *Bacillus thuringiensis* (Bt), since it already exists in nature, infecting 52% of the world’s arthropods [93], rather than as a genetic-drive system which would face heightened regulations. *Wolbachia*-based strategies have not been categorized in a regulatory sense as genetic modification, which has offered some benefits in terms of implementation, but categorization of *Wolbachia* as a biopesticide is not always an advantage. Surveys of communities in Australia, probed before application of *Wolbachia,* showed that communities can be sensitive to the risks of “biocontrol” citing a number of locally known cautionary tales [94]. Distinction of applications of *Wolbachia* as a biopesticide as opposed to a genetic mechanism and specifically a gene drive is not precise, since *Wolbachia* does drive a set of genes into mosquito populations (the entire bacterial genome) that was previously foreign, the endosymbiont is maternally inherited and integrations of *Wolbachia* genes have been identified in many *Wolbachia* host species (including *Ae. aegypti*) which is perhaps not surprising given that the bacterial genome is heavily occupied by repeats and transposable elements, [95,96,97,98,99,100,101,102].

### 1.4. Synthetic Approaches to Genetic Strategies for Mosquito Control

Synthetic genetic technologies have a number of advantages over technologies employing only existing biological systems. The components of a synthetic construct are relatively small, their functions are more fully known and the site in the mosquito genome where they are located can be characterized, such that modifying the pathogen-targeting or mosquito suppression genes could be relatively straight-forward. It follows, then, that the effectiveness of a defined set of synthetic genes could be more accurately assessed for aspects that need improvement, and improvements more easily engineered. In contrast, the biology around dengue resistance and cytoplasmic incompatibility phenotypes incurred by *Wolbachia* infection are largely unknown; this paired with our current inability to transform the endosymbiont means that we cannot currently adjust the approach at the genetic level. A given genetic strategy requires an effector gene or a set of genes to encode population suppression or pathogen-blocking genotypes and a method to detect whether a mosquito bears the effector genotype (e.g., a marker). Modular designs for genetic constructs allow the combination of promotors, effectors and markers that can encode a set of gene modules that can execute a very specific purpose into a single genetic package. These designs are straight-forward to construct once appropriate genetic elements exist and are characterized for each module.

#### 1.4.1. The “Flightless Female”: An Illustration of a Fully Realized Population Suppression Strategy Using Synthetic Gene Modules

Genetic engineering of synthetic phenotypes has led to the development of population-suppression strains of mosquitoes, the most successful so far is known as the “flightless female” *Ae. aegypti*. This strain contains a genetic element that encodes a toxin to destroy the wing muscles of females. Without normal function in wing muscles, the females are unable to mate or search for food and oviposition sites. The transgene construct responsible for this phenotype encodes a repressible, late-acting, sex-specific lethal, allowing rearing and sexing prior to release as well as survival of larvae in subadult stages to compete with wild larvae [103,104,105,106]. The gene set that encodes these phenotypes is spread into the population by male carriers who are unaffected by the transgene. Males are being released as a control strategy in Brazil and Florida after large cage trials validated their usefulness for population suppression [107,108]. Similar strains have been developed in *Ae. albopictus* and *An. stephensi*, but have not yet been utilized to control wild populations [109,110]. These strains offer a population suppression strategy in species where no SIT or SIT-like strategy has yet been successful.

The combination of gene modules used to generate this line illustrate the utility of a synthetic construct in encoding marker and effector genes with expression characteristics that serve a specific genetic mosquito control strategy. An accounting of the requirements for the control strategy dictates the modules needed. *A lethal phenotype:* the effector gene *VP16* causes cell toxicity [111,112]. *Female specificity:* ideally, the lethal gene would affect females and not males, since females transmit pathogens and males can be carriers of the genes to wild populations. A female specific promoter, *AeActin4*, is not expressed in males, but drives expression of VP16 in the female flight muscles. *Repressibility of lethality for rearing*: viable females are needed, so the construct was designed to be repress *VP16* expression using tetracycline, in which the mosquitoes can be reared in the lab, but since they aren’t exposed to tetracycline in the wild, the effector will be turned on in wild offspring of transgenic mosquitoes. *Identification:* To distinguish between transgenic and wild-type mosquitoes, a gene for the fluorescent marker, *DsRed*, is encoded on the construct and expressed in the eyes of the mosquito. Each of the components of this system stems from previously identified characteristics in insect biology, combined intelligently to achieve a very specific purpose. One would be hard-pressed to identify a naturally occurring biological system that meets all of these requirements. It’s important to note here that the more we understand about mosquito biology and the biology of other insects, the more power we have to design a construct that most serves a given purpose. This brings us to the benefit of being able to specifically study genetic systems in mosquitoes; molecular tools to study basic biology are needed to support the applied science of elimination and eradication of vector-borne disease.

#### 1.4.2. Synthetic Engineering for Complete Pathogen Blocking

Selection for mosquitoes that are resistant to human pathogens was recognized early on as a disease control strategy that would take advantage of genes already in mosquitoes [23,27]. Some candidate genes and pathways have been identified in *An. stephensi, An. gambiae* and *Ae. aegypti* as potential candidates for such efforts [113,114,115,116,117,118,119,120,121,122,123,124,125,126]. For example, perturbations of AKT signaling and transgene-mediated expression of the IMD signaling gene Rel2 have both been demonstrated to increase the ability of *An. stephensi* to kill *P. falciparum* [114,127,128,129,130]. Increased dengue resistance was demonstrated in *Ae. aegypti* with silencing of vATPase and IMDPH expression [117]. Some synthetic genes constructs have been successful in conferring pathogen-resistant phenotypes. The first demonstration of a stably-integrated synthetic construct that produced an anti-pathogen product in mosquitoes was reported in 2002 with the generation of a transgenic *An. gambiae* line expressing a peptide that bound to *P. berghei* [130]. Other constructs have since been developed in both *Anopheles* and *Aedes* species with increasing levels of pathogen resistance (for a comprehensive list see Table 13.2 in [131]), including lines in both *An. stephensi* and *Ae. aegypti* that completely eliminated pathogen presence in the salivary glands, the tissue it must reach to be transmitted to humans [132,133]. In these synthetic cases, defined biological components are combined in a way that meets a specified goal: promoters specific to that species and expressed locally and temporally consistent with pathogen exposure, gene products that inhibit a pathogen, and a visual marker of the transgene.

### 1.5. A Technology-Driving Need for Site-Specific Gene Editing

Technological improvements in mosquito genetics in the 20th century and into the following decade had yet to sufficiently address an important gap: we still lacked the ability to target a specific site on a mosquito genome either for probing biological functions, for the targeted insertion of a synthetic construct, or for a gene drive. While transposons were successful platforms for transformation, their short target sites allow them to insert into many locations in the genome [134,135]. For the construction of transgenic lines, this means that the site in which a synthetic construct is inserted could cause a fitness reduction in a transgenic line, depending on where they insert [136,137,138,139]. For gene drive based on the mobility of transposons, it means that the drive would likely incur a fitness cost as a result of continued re-mobilization into new sites in a genome. The mechanism of HEGs for gene drive were based on site-specific engineering, accomplished by target recognition and endonuclease functions of a HEG, but engineering a HEG to target a specific nucleotide sequence is not straight-forward [140]. The successful targeting of a HEG to a mosquito locus was reported in 2007 [141] and a HEG-based gene drive was reported in 2011, almost a decade after they were proposed as a basis for a gene drive mechanism [79]. The application of ϕC31 to mosquitoes improved our ability to move constructs into known locations in the genome, but these “docking sites” were pre-established in transgenic lines by random transposon-mediated integration, so specific genes could not be targeted for modification (Table 1) [50,132,142,143].

The application of zinc-finger nucleases (ZFNs) to gene targeting introduced a major improvement to genetic studies in mosquitoes. Zinc-finger domains recognize the shapes of nucleotide triplets in the major groove of a DNA double-helix and could be engineered to recognize a 18 nucleotide sequence such that a whole array of protein effectors could be recruited to a very specific site in the genome [144,145,146]. Zinc-finger domains are conjugated to a FokI type II restriction endonuclease and created in pairs recognizing sequences flanking a target-site so that a pair of ZFNs would create a double-stranded break at a specific genomic locus [146,147]. These improvements meant that engineering target-site recognition with ZFNs was modular and more-straight forward than for HEGs. However, the cost of a ZFN and the low success rate was still prohibitive to the technology being used by most labs for biological studies. Finally, in 2010, a system that was affordable and could be engineered in-house, made targeted mutagenesis accessible for molecular biology labs: transcription activator-like effector (TALE) nucleases or TALENs. Like ZFNs, TALENs are modular, could be encoded on a plasmid by cloning and were relatively efficient [148]. The recognition of each nucleotide on a DNA target was encoded in the 12th and 13th amino acid of each 34 amino-acid repeat; a peptide stretch of 18 or 19 repeats could be engineered to recognize any nucleotide sequence and when conjugated to the FokI domain and could induce site-specific DNA cleavage [149,150]. While 18–19 repeats in the TAL portion of protein initially made cloning difficult, kits were soon developed to do this easily and by 2013 a library of TALENs existed for targeting 18,740 human protein coding genes [151]. Gene-editing in *Ae. aegypti* and *An. stephensi* using ZFNs and TALENs were reported in 2013 [152,153,154]. With increased efficiency, an order of magnitude difference in price, and less difficulty in constructing TALENs in the lab, TALENs were more accessible than previous gene-editing approaches. But the timing of TALEN development was almost concurrent with the leveraging of CRISPR/Cas9 biology for gene-editing, meaning that TALENs usefulness was short-lived.

Within the 2003 proposal to utilize HEGs as platforms for gene drive, Burt highlighted the condition that would bring ease and efficiency to site-specific engineering; the homing mechanism of group II introns is specific DNA targeting by Watson-Crick base-pairing, which is straightforward to reprogram, instead of by a specific protein shape, as is needed for HEGs, ZFNs and TALENS [75,155]. Group II introns were not pursued for gene drive because of other limiting complexities, but the bacterial CRISPR-Cas systems that also use base-paring for DNA recognition were harnessed for site-specific gene-editing, providing by far the most-successful basis for the development of synthetic gene drive systems to date.

## 2. What Cas9 Has Done for Synthetic Genetic Engineering and Gene drive

Shortly after Cas9-mediated gene-editing was introduced in 2012 [156], and first demonstrated in human and mouse cells in 2013 [157,158], the technique was applied to mosquitoes to create specific gene-mutant *Ae. aegypti* lines in 2015 [159,160,161,162]. In the same year Cas9 was used to develop the first efficient insect gene drives in *D. melanogaster, An. stephensi* and *An. gambiae* [163,164,165]. Since 2015 we have also seen an expansion in the number of mosquito species to be genetically modified to include *An. funestus*, which did not have transgenic technologies available before the introduction of Cas9-mediated editing [166]. The reader will appreciate how quickly these applications arose following the introduction of CRISPR/Cas system to genetics, given the history in genetics advancement reviewed thus far (Table 1).

Cas9 is an endonuclease borrowed from the CRISPR biology of the bacteria *Streptococcus pyogenes*. CRISPR stands for “Clustered Regularly Interspersed Palindromic Repeats” and refers to the loci that are widespread in bacterial and archaeal genomes that store sequences of parasitic nucleic acid to which they were previously exposed [167,168,169,170,171]. These are co-located on the bacterial genome with *Cas* (CRISPR associated) genes which are expressed and used to target and destroy incoming parasites with homology to small RNAs derived from the CRISPR loci. Cas9 endonucleases can be targeted to virtually any region of a genome by encoding a homologous ~20 nucleotides on a modified single guide RNA (sgRNA), which will localize Cas9 to the target site for double-stranded cleavage of the DNA [156].

The application of Cas9 to gene editing in mosquitoes has made mosquito gene-editing cheaper, more targeted and more efficient. Cas9-nuclease targets a specific location using a sgRNA which is easily programmed using a $20 oligonucleotide. The Cas9 protein can be ordered or made in-house (<$200 for bottle large enough to inject >10,000 embryos) and mixed with in vitro transcribed sgRNAs targeting a desired genomic locus. This is an improvement over TALENs and ZFNs, which required novel protein engineering for each target. Cas9/sgRNA complexes are delivered to insects by embryo injection (these embryos represent the Generation (G) 0); for *Ae. aegypti*, this has resulted in gene-specific mutation efficiency as high as 90% in G_1_ offspring [172]. However, mutations with non-visible phenotypes are not easily detected and maintained without the insertion of a marker. This can be achieved by co-injection of a donor plasmid that encodes a marker (e.g., fluorescence in the eyes or body) between regions of homology flanking the sgRNA target site.

The donor provides a template for the mosquito cells to repair the Cas9-induced double-stranded break by homology-directed repair (HDR) instead of non-homologous end joining (NHEJ). Initial attempts at Cas9-mediated transformation of *Ae. aegypti* demonstrated efficiencies 0.1–0.4% of injected G_0_ embryos [161]. This efficiency is lower in *Anopheles* species. Cas9-mediated transformation is less than 0.01% in *An. stephensi* and in *An. gambiae,* pre-establishment of transgenic lines expressing Cas9 and sgRNAs are generally required for transformation [164] (Andrea Smidler personal communication). Improvement has been made with the demonstration that stable integration of a construct expressing Cas9 in the germline can increase mutation and integration efficiencies; application of germline Cas9 expression in *Ae. aegypti* has increased efficiencies of targeted gene integration to ~2% [172]. These efficiencies are still low, but the advantages of the system make it the leading technology for gene-editing in mosquitoes; Cas9-mediated editing can be employed in reverse genetic studies of gene-function, which wasn’t possible with transposon- and ϕC31-based transgenics and it is more adaptable, cheaper and technically more straight-forward than using ZFN and TALENs.

Cas9-mediated gene editing has also provided a platform to develop an efficient gene drive. A Cas9-based gene drive encodes the Cas9 endonuclease and an sgRNA targeting the homologous location on a chromosome that doesn’t bear the construct. The Cas9 and sgRNA genes can be linked tightly with effector genes that confer either anti-pathogen or suppression phenotypes. When the Cas9 and sgRNA are expressed and target the homologous chromosome the entire cassette including the effector genes will be copied into the homologous site when the chromosome bearing the gene drive cassette is used by the cell for homology-directed repair. It has been demonstrated that at least ~17 kb of cargo can be encoded on the cassette and effectively copied during HDR [164]. Under these circumstances HDR is much more efficient than is seen during initial transformation; 90–100% of offspring contain integrations of a gene cassette indicative of repair by HDR.

**Figure 1:**
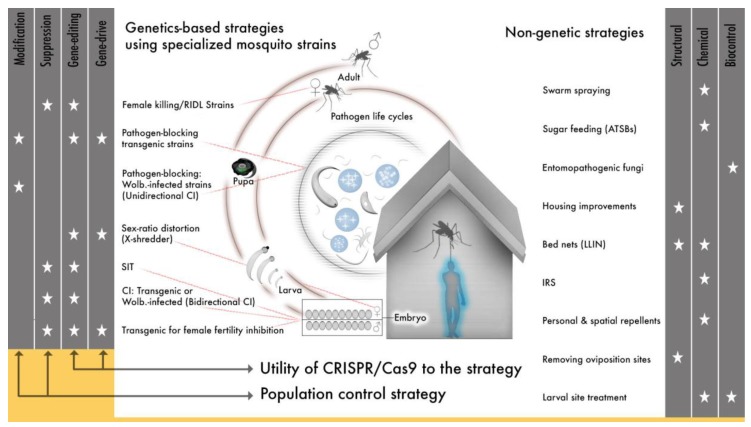
Genetic approaches to vector-borne disease offer a substantial complement to the existing tool-set. A scheme illustrating the contribution of specialized mosquito strains, including both transgenic and *Wolbachia-*infected mosquitoes, to vector control. Each strategy is categorized on the right as modification or suppression, whether the strategy can utilize Cas9 gene-editing or gene drives are indicated with a star. Whether a specific mosquito life-cycle stage or the pathogen life-cycle are targeted is indicated by a red line. The center schematic is a stylistic representation of vector-borne pathogens and is included to illustrate where specialized mosquito strains can be used to inhibit pathogen development in the mosquito. A summary of existing, non-genetic tools is to the left of the schematic. RIDL: release of insects carrying a dominant lethal, SIT: sterile insect technique, CI: cytoplasmic incompatibility, LLIN: long-lasting insecticide treated nets, IRS: indoor residual spraying, ATSB: attractive toxic sugar baits.

The original bacterial Cas9 could potentially modify any arthropod genome, a quality that led almost immediately to the application of gene drive to multiple mosquito species and the expansion of transgenic technologies to new dipteran and non-dipterans insect species [166,186,187,188,189,190,191,192,193,194,195,196,197]. The species specificity of SIT and synthetic transgenic drives offers an advantage over less targeted vector control approaches (e.g., insecticides) in that control can be applied to a specific insect species and can avoid beneficial and neutral insect species or non-insect species. But this same quality means that an individual genetic approach will have to be employed for every target species. This can be a major limitation for mosquitoes, if the technology to develop individual gene drives is not efficient since, in many regions there are several mosquito species that are recognized as important vectors, many more that may be under-recognized vectors and because *Anopheles* species exist as morphologically indistinguishable, but genetically distinct cryptic species [198,199,200,201]. Cas9 gene-editing (and similar emerging platforms) offer a technically realistic means to target several species engineered genetically to serve either modification or suppression strategies that can be integrated with other approaches to help address a need for more vector control tools in light of evolving resistance to many of our current approaches (Figure 1).

## 3. A Dramatic Shift in the Application of Genetic Approaches to Mosquito and Mosquito-Transmitted Disease Control: Perspectives on the Future

“*Clearly, the technology described here is not to be used lightly. Given the suffering caused by some species, neither is it obviously one to be ignored.*”

—Austin Burt [75].

The application of Cas9 to gene drive represents a second major shift in the field of vector genetics for disease control. It is now technically straight-forward to develop any number of genetic strategies and so add a substantial new subset of tools to vector control efforts (Figure 1), so how do we use CRISPR/Cas9 and similar technologies for gene editing and gene drive given the enormous potential to alleviate human suffering? Challenges will arise in efforts to implement molecular techniques developed in the lab to field releases. We will do well to heed the lessons learned by previous work and to carefully consider the challenges that have emerged alongside the introduction of efficient, site-specific gene-editing.

### 3.1. Long-Standing Considerations

#### 3.1.1. Early Successes Provide Templates for Predicting Success

Sterile insect technique and genetics-based control tools were first tested in mosquito populations in the 1960s. Initial field trials with *An. quadrimaculatus*, *Ae. aegypti* and *Cx. pipiens fatigans* failed to control test populations and it was only when chemosterilization with the chemical agent thiotepa replaced radiation as the primary means to sterilize males, that the switch in methods led to the first successful mosquito SIT program [202,203,204,205,206]. Chemosterilized *Cx. pipiens* eliminated the native population after 10 weeks of sterile male releases in Seahorse Key (FL, USA) [202]. These early successes and failures suggest that vector control tools that look promising in the laboratory don’t always translate into promising outcomes in the field. Better means to predict field success are needed. Compared to laboratory studies, wild populations may be more likely to experience density-dependence and the effect of released sterile males could be reduced by migrating inseminated females from outside of the release area [203]. Early modeling efforts to predict the success of sterile male release programs suggested that even modest immigration of mosquitoes into a release area could doom a population suppression program to failure, leading Asman and others to conclude that “with immigration, eradication is not possible and continued sterile male release would be required in order to maintain tolerable low levels of the pest” [200]. In contrast, population modification strategies are likely more resilient to immigration of mosquito populations into and out of a release area and the replacement rate can be predicted by the fitness differences between the released mosquito and wild-type [203]. Although gene drive mechanisms exhibit non-Mendelian inheritance patterns and can likely tolerate more fitness costs than mechanisms with Mendelian inheritance, fitness still plays an important role in predicting success of population suppression or modification [139,207,208,209].

Not surprisingly, we can still largely depend on a 50-year-old framework for assessing the potential of a mechanism prior to implementation, considerations for release and evaluating the outcomes. Perhaps the most important appeal to be made of researchers is to aggressively collect data. Recommendations that data be collected on the disease and vector biology, environment and population dynamics of the targeted insect, the fitness and thus competitiveness of released strains compared to wild populations, and the rearing and handling requirements strategy are in every milestone assessment review on the topic of genetic control of insects and cannot be underemphasized in the context of gene drive [20,23,53,210,211,212,213,214]. The drive rate, tolerable fitness reductions, tolerable levels of drive-resistance, and optimal distribution and timing for successful release need to be modeled with field-relevant parameters to predict success of a release program. Ongoing surveillance data will be critical to interpreting the success of release strategies, identifying useful modifications to an approach including replacement of a drive-strategy, and determining whether a reversal of the drive is necessary.

#### 3.1.2. Identify and Measure Appropriate Proxies for Assessing the Fitness of a Treatment Strain

Understanding the contribution of the fitness of specialized mosquitoes relative to the wild target population is pivotal to predicting the success of a genetic strategy for vector control, since the transfer of genetic material from released mosquitoes to offspring in the wild depends on competition with wild mosquitoes for resources and mating. Most genetics-based control strategies are first tested in cage trials, where population modification or suppression can be observed under different ratios of genetically modified individuals to unaltered individuals, and under conditions where life history traits can be carefully measured and environmental variables such as resource availability, temperature and humidity can be controlled. Standard laboratory assessments prior to field release of a genetically altered insect include measuring mating competitiveness, but rarely are more holistic measurements of fitness captured [214,215]. While unsuccessful cage trials can signal when a technology will likely fail in the field, a successful cage trial does not always translate into field success. Even within the laboratory, trial-to-trial variability is sometimes only explained by looking at fitness parameters across the insect lifespan. In 1973, trials with *Drosophila* testing population replacement of a laboratory population with modified strains that had compound autosomes, demonstrated that predicting successful versus unsuccessful strains required more holistic estimates of fitness than egg hatch rate alone [211]. Similarly, failed field releases of irradiated *Ae. aegypti* males for population suppression in Pensacola, Florida was later attributed to poor survival in the pupal stage, again highlighting the need for thorough evaluation of the consequences of sterilization or genetic-alteration on mosquito fitness across life stages prior to release [205,214]. Failed release programs have the consequence of not only wasting resources, but threatening the success of future programs by creating public mistrust or donor reluctance in supporting these types of technologies.

Evaluating fitness costs of gene drive technologies involves estimating fitness by measuring life history traits across the mosquito life cycle, or tracking allele frequencies in populations over time. Both approaches are difficult. The best fitness proxies to estimate mosquito fitness have not been clearly identified, but life history theory suggests that fitness is more sensitive to life history traits that describe early life events such as egg hatching rates and larval survival than to late-life traits such as adult survival. Juvenile traits should therefore be prioritized as the most predictive life history traits in estimating fitness when evaluating the potential for a gene drive to spread. Once spread, the impact of the drive mechanism on mosquito fitness can be used to understand potential evolutionary responses of mosquitoes to the drive mechanism and co-evolutionary changes in the parasite populations they spread. Even a drive mechanism that comes with minimal fitness costs to the mosquito could fail to reduce disease incidence or prevalence if it affects vector competence while only minimally changing vectorial capacity [216]. Understanding both vector and parasite fitness traits and their potential to evolve in response to genetics-based technologies should be understood prior to releasing these technologies into the field. A combination of computer simulations, laboratory studies that evaluate fitness parameters across the mosquito lifespan, an understanding of local ecology, and ongoing surveillance after release programs begin will be important to a robust deployment of genetics-based control.

#### 3.1.3. The Importance of Modeling

Modeling allows us to integrate large amounts of collected data and logically draw conclusions that are not always intuitive to predict success or failure of a release program. This is especially important for identifying the ability of a genetic approach to work across different mosquito-pathogen systems. Models demonstrating the potential of certain gene drive strategies have given us reason for both optimism and concern [210,217,218,219,220]. Working from a perspective that different drive approaches will be optimal for different applications, Eckhoff modeled the vector and drive mosquito population outcome using different gene drive strategies in specific locations (Tanzania and Garki) with varying homing rates and fitness reductions [217]. Optimism for gene drive is supported by their suggestions that even the most challenging situations stand to benefit from a gene drive strategy if a certain set of qualifications are attained. With very high homing rates demonstrated for recently developed drives, it is realistic that such qualifications can be met, even with a fitness reduction in transgenic drive insects [217]. Eckhoff identified major unknowns in their studied locations including aestivation behavior of vectors and movement of males, and differences between such behaviors between vector and transgenic drive insects that would inform the success of a release program. Since multiple vectors and species complexes contribute differently to disease transmission, modeling will also serve to inform which species are most important to target in a given region in order to have the greatest impact, which will in turn inform the lab-based efforts to develop appropriate drives for each species.

#### 3.1.4. Which Tools, Where

The complexity of vector-borne disease transmission dynamics across the globe means that a successful global eradication strategy, a current goal for both malaria and lymphatic filariasis, will require a diverse tool set and region-specific vector control application of a subset of those tools [221,222,223]. The current toolkit includes insecticide-treated bed nets (LLINs), anti-malarial and anti-filarial drugs, a yellow fever virus vaccine, indoor residual spraying, structural modifications to housing (improved housing material, screens, eave-tubes), larval habitat treatment, *Wolbachia*-based gene drive (Eliminate Dengue), *Wolbachia-*based population suppression (MosquitoMate and Verily), and a transgenic RIDL strain (release of individuals carrying a dominant lethal, flightless female strain OX513A) (Figure 1). This toolkit is growing to soon include additional arbovirus and malaria vaccines. Some tools, specifically those dependent on pyrethroids and other insecticides, are becoming less useful due to growing insecticide-resistance [88,224,225,226,227,228]. The variety of genetic tools currently in development since the advance in genetic technologies can target many aspects of mosquito and pathogen life cycles and have real potential to improve this toolkit (Figure 1). Theoretical analyses that have evaluated added benefits from combining vector control tools have suggested that some combinations are more effective than others. For example, LLINs in combination with larvicides are predicted to result in greater reductions in mosquito abundance than the use of LLINs with IRS or LLINs alone [229]. In practice, combined use of BT with LLINs has reduced the incidence of malaria infection to a greater extent than LLINs alone in western Kenya [230]. Similar analyses and field experiments should be done to evaluate how genetic technologies will work in combination with other existing vector control measures; the inclusion of newly available and/or underutilized technologies in these analyses will greatly benefit our ability to achieve local elimination and global eradication goals. Specifically, identifying the vector control approach or approaches that most greatly impact disease transmission will provide useful direction in releasing specialized mosquito strains (transgenic or *Wolbachia-*infected) in areas where they best complement current approaches and have maximal effect in reducing disease.

### 3.2. New Considerations

#### 3.2.1. Gene drive Resistance

New considerations regarding the potential for drive-resistance have arisen from the molecular mechanisms of site-specific endonucleases. Homing endonucleases- and Cas9-based gene drives generate resistance because the double-stranded break necessary for HDR-mediated insertion of the drive construct is occasionally repaired by using non-homologous end joining (NHEJ) instead. Repair by NHEJ often causes insertions or deletions in the target site, rendering it impervious to future targeting by the endonuclease. When HEGs were first proposed as a site-specific gene drive, it was recognized that variation and mutation in the sequence of the target site would provide a source of resistance to the drive, but also that strategic drive design would likely lead to successful gene drive [75,231]. Recent modeling of sources of sequence variation, both naturally occurring and induced by the drive mechanism itself, suggest that resistance will almost certainly emerge to a simple one-target Cas9-based gene drive [219]. Even modest rates of NHEJ are likely to impact the effectiveness of gene drive strategy [217,219,232,233]; in order for a gene drive to be successful, the emergence of resistance to the drive must be at least five orders of magnitude lower than what have been observed in recently demonstrated drives [163,164,165,179]. Gene drives based on site-specific endonucleases rely on the repair mechanism of the organism, so that it may be difficult to engineer a gene drive scenario that manipulates that host-biology such that HDR is favored over NHEJ to such an extent as to provide orders of magnitude fewer occurrences of resistant alleles. Approaches to mitigate the effects of gene drive resistance have been suggested, including targeting multiple sites for disruption, targeting highly conserved sites or sites where indels caused by NHEJ would cause lethality or infertility, and altering the promotors and genomic targets of a gene drive. Recent investigations into these suggestions provide optimism that shortcomings of single-target Cas9-based gene drives can be overcome in future gene drive designs [179,232,233,234,235]. Multiplexed gene drives are expected to substantially decrease the likelihood of gene drive resistant alleles emerging using only a few additional targets [232]. Feasible ways to multiplex Cas9-based gene drives have been demonstrated using post-transcriptional processing of several sgRNAs expressed from a single promoter, but these have not yet been applied to mosquitoes [232,234,236,237,238].

Both *Wolbachia*-based and transgene-based approaches to vector control, including MEDEA- or transposon-based drives and transgenic population suppression strains, face the risk of becoming inert due to effector inactivation. Some data suggests that mosquito immune responses to *Wolbachia* prime the mosquito against viruses, like dengue, but this response could weaken over time as *w*Mel and *Ae. aegypti* co-evolve, dampening refractoriness to RNA viruses [239,240]. *Wolbachia-*based population modification targets a rapidly evolving virus, which could change to avoid the mechanism causing refractoriness in *Wolbachia*-infected *Ae. aegypti*. Because the biology of these interactions is largely unknown, it is prudent to closely monitor the refractoriness of mosquito populations that have been modified with *w*Mel infection. Concern has been raised that *Ae. albopictus* populations targeted for suppression using *Wolbachia* cytoplasmic incompatibility phenotypes could become resistant to the suppression mechanism, derived from demonstrations that mosquitoes that have low abundance of the natural strains of *Wolbachia* are not completely sterile when they mate with *w*Pip infected mosquitoes [241]. Such mosquitoes do exist in the targeted populations and depending on how many *w*Pip-infected females escape sexing during rearing, *w*Pip may become established in the wild population, rendering the strategy useless [241]. However, recent developments in sex separation techniques will likely improve the proportion of females released to address this issue [226]. Transgenes and transgenic gene drives could face a different type of inactivation. Similarly to *Wolbachia*, pathogen blocking could become ineffective because of pathogen evolution. Transgenic approaches have an advantage over *Wolbachia* in this case because the biology of pathogen blocking is known and encoding numerous targets for the pathogen can reduce the development of pathogen resistance to products of the transgenic construct. Alternatively, a transgenic drive mechanism could become unlinked from the effector genes, so that the drive continues, but the pathogen blocking alleles could be lost from the population; this can be mitigated by thoughtful arrangements of the drive and effector components of a synthetic construct such that the drive becomes inactivated if the construct is disrupted by recombination [70]. Further, we know that many organisms inactivate selfish genetic elements using the piRNA pathway. While this has not been examined in mosquito transgenic lines the component of these pathways do exist in mosquitoes [242,243,244] and examples of transgenic constructs becoming piRNA producers with inhibited gene expression exist in Silkworm cell lines and in *Drosophila* [245,246,247,248,249,250]. We appeal to the broader vector biology field to study the underlying processes of the *Wolbachia*-mediated virus refractoriness and transgene inactivation and at the risk of being redundant are hopeful that these biological processes will be easier to probe with the application of reverse-genetics by site-specific gene editing.

#### 3.2.2. Off-Target Effects of the Cas9/sgRNA Complex

Potential off-target activity of the Cas9 due to homology between sgRNAs and non-target sites in the genome presents an interesting challenge for Cas9-based gene drive. Off-target effects are not a Cas9 specific concern: RNA interference (RNAi) methods utilizing the micro- and small interfering-RNA (miRNA and siRNA respectively) also rely on ribonucleoprotein activity and are prone to inhibiting transcripts other than the intended target. An understanding of ribonucleoprotein complex characteristics and homology-based cleavage requirements have enabled researchers to faithfully use RNAi in genetics, but similar considerations for optimizing sgRNAs for use with Cas9 have not been completely teased out. To date unintended mutations induced by Cas9 have been difficult to detect and quantify. In general, genome sites with potential for off-target cleavage by a given sgRNA/Cas9 complex can be predicted using computational algorithms and then probed for mutations by PCR-based assays or larger units of genetic material (DNA or cDNA) can be deep-sequenced for indels or single nucleotide polymorphisms (SNPs) [251,252]. However, Cas9-induced off-target SNPs can be difficult to quantify accurately without a robust dataset documenting all SNPS already existing in a population to reduce false identification of off-target mutations. In mosquitoes, as in other organisms, efforts have not led to any detectable, off-target Cas9-induced mutations but a thorough analysis in mosquitoes has not yet been reported. Efforts underway by the *An. gambiae* 1000 genomes project to build a genome data set from wild-caught mosquitoes will aid in detecting potential off-target effects [159,191,253,254,255]. Other methods for detecting off-target activity of Cas9 include assays for identification of double stranded breaks or genome binding of a Cas9/sgRNA complex with inactivated nuclease activity, though these methods have not yet been used in mosquitoes [256,257,258]. Rare unintended mutations have been identified in mouse studies and notably, off-targeting in in vitro studies has been more highly reported, likely because many more genomes exposed to the Cas9/sgRNA complex are sequenced, giving the researchers a higher power to detect very rare off-target mutations [252,257,258,259,260,261].

The impact of unwanted mutation for gene drive applications is higher than for in-lab gene editing since mutations arising after release of a gene drive into a population have the potential to persist. In the case of a gene drive that is self-limiting, like those proposed for population suppression, the mistakes would be eliminated with the population itself. Off-target mutations are another source of potential fitness effects in gene drive mosquitoes designed for population modification strategies. These considerations fall squarely under the category of “unintended consequences” of the release of transgenic organisms with a drive mechanism, a common topic in academic, political and lay discussions on the utility and ethical considerations of gene drive. It thus behooves us to be able to quantify and model the possibility of introduction of mutations derived by off-targeting of the drive components into the target mosquito populations. This will require a thorough sampling of the genomes that exist in the target population and continued sampling of genomes after release of gene drive laden mosquitoes with the specific intention of detecting new, unintended mutations.

It may be that such quantification will provide an increased confidence in the Cas9-based gene drive strategies already in development. However, should prudence require increased specificity in nuclease activity, approaches to increase specificity and efficiency of the RNA-guided endonuclease have already been demonstrated to be effective, including truncation of the sgRNA, the use of Cas9 nickases (a Cas9 mutant that only cuts a single DNA strand) with offset target sites, highly-specific Cas9 mutants like eSpCas9 and SpCasp-HF1 and alternate RNA-guided nucleases like Cpf1 that create sticky double stranded breaks [251,257,262,263,264,265,266,267]. Algorithms for sgRNA design are updated regularly to incorporate both new knowledge on off-target effects and increasing number of genomes in order to generate specific and efficient sgRNAs [268,269]. The measuring and addressing off-target effects and their potential impacts on gene drive strategies are areas that will continue to require a substantial amount of attention.

#### 3.3.3. Containing an Efficient Gene drive

The likelihood that a gene drive cassette would persist in the environment in the case of accidental release from lab containment, a test site or a rearing facility has been modelled between different drive mechanisms and depends variably on the number of mosquitoes released and the efficiency of the drive [270]. The novelty of the levels of efficiency observed for recent applications of CRISPR/Cas9 biology requires a re-consideration of the potential to control the activity of the gene drive both during experimentation in the lab with the possibility of accidental release and once intentionally released into the wild for disease control applications. A high level of care when working with gene drive constructs has been echoed through the field as essential to the ethical development of the CRISPR/Cas9-based technology for all potential applications [235,271,272,273]. In addition to gene drive containment recommendations, several mechanisms to reverse a drive following the intentional releases are being developed. These strategies are particularly relevant for population modification strategies, since population suppression strategies are inherently limited. The Cas9/sgRNA complex and HEGs have both been proposed as the basis of both reversal mechanisms including designs for synthetic constructs driven *in trans* by another construct, even in several iterations (called “daisy chains”), in order to limit the spread of independent self-driving constructs in the case of an accidental release [75,152,274,275]. Such systems have been demonstrated in yeast and modelled to provide a robust and containable drive mechanism [275].

#### 3.3.4. Globalization

Globalization impacts vector control strategies in two foreseeable ways. First, mosquitoes released for population suppression and replacement efforts disperse without regard to political boundaries. In an age of increasing global movement by humans, dispersal of genetically-altered mosquitoes outside of intended release zones is inevitable. Genetics-based control efforts will have to evaluate how to contain genetically-altered mosquitoes in population replacement programs, and deploy intensive surveillance for population suppression efforts to avoid migration into and out of the release zones when there are concerns over unintended consequences. For suppression programs, migration into a release area or an area that has already achieved local elimination could result in repopulation of the mosquitoes we were trying to control. For population modification strategies, dispersal of released mosquitoes outside of intended areas could create political tensions with bordering countries that may not have approved the technology. Improved molecular tools, buffer zones with alternative control strategies near political borders, remote sensing of mosquitoes with improved traps or acoustic identification technology [276], or citizen scientist campaigns to detect or sample local mosquitoes could all contribute to more effective and contained vector control campaigns. To address concerns of population replacement campaigns resulting in new populations susceptible to new diseases, improved xenosurveillance and future efforts to make surveillance faster and easier will likely make any unintended consequences quickly detectable and easier to address [277].

Second, rapid globalized human communication is a novel tool for complex vector management approaches and can be leveraged to integrate the various levels of information that will influence the planning, implementation, assessment and adjustment of a strategy, including information on the population structures of target regions, current release efforts, surveillance of specialized mosquito strains following release and resulting epidemiological changes. Many release strategies are being tested around the world, with only minimal available information in these categories. Eliminate Dengue has a map indicating sites where *w*Mel-infected mosquitoes are being released with links to the progress made in those areas. Oxitec and MosquitoMate have information about ongoing releases of transgenic line OX513A *Ae. aegypti* and *w*Pip-infected *Ae. albopictus* respectively, but without interactive maps [227,278]. Verily Life Sciences will begin releases of *w*AlbB-infected *Ae. aegypti* in California and Queensland soon. Releases of sterile *An. arabiensis* are ongoing in South Africa and Sudan [279,280]. Test releases of sterilized *Ae. aegypti* have been conducted in Italy, Indonesia, Mauritius and China and, *Wolbachia*-laden *An. polynisiensis* and *Cx. pipiens quinquefasciatus* are being tested in for cytoplasmic incompatibility in French Polynesia for lymphatic filiariasis control and La Reunion for arbovirus control, respectively [281]. Optimally, data collected in concurrence with these releases could be integrated into a single framework. VectorBase.org is particularly well poised to undertake these efforts; vector genetic information contributed by scientists is integrated and openly available to others. Mosquito insecticide resistance is being collected in this database, is accessible also as interactive map, and is being expanded to include additional phenotypes of existing mosquito populations [282]. Further expansion including transgene information and *Wolbachia-*infection status overlaid with epidemiological data such as those presented by internet disease surveillance sites like healthmap.org and the Malaria Atlas Project will support release and data openness goals [283,284]. Internet-based platforms for the integration of population and epidemiological data and ongoing releases could promote the sharing of information between scientists, practitioners and policy makers and encourage an atmosphere of participation and openness among lay people.

## 4. Conclusions

The technical innovations that have led to efficient gene drives have caused a shift of focus in the field of vector biology from tool development that might allow us to use genetics to combat disease vectors, to considerations, experimentation, and modeling of what it will look like to use these approaches effectively. Many considerations for successful implementation of genetic tools for vector control have been on the minds of biologists for more than 60 years and some have arisen from the specific tools that we intend to use. The breadth of molecular mechanisms and the adaptability of genetic engineering afforded by the CRISPR/Cas9 and related systems means that we have many avenues to pursue the engineering of strains that address the complexities of different control strategies and that can be integrated with existing structural, chemical and biocontrol tools for successful vector-borne disease programs. In light of a need for unique sets of tools under different environmental, vector and pathogen circumstances, we should not merely undertake to prove superiority of the technology our own group is developing, but instead to provide quality analyses of the purposes of and the circumstances under which our tools will and will not be useful, so that a community adding a genetic component to their disease control strategy can make informed decisions on what will have the greatest epidemiological impact.

## Figures and Tables

**Table 1:** Comparison of genetic techniques and their application to mosquito genetic manipulation and gene drive.

	Genetic Techniques					
	Transposons	ϕC31	HEGs	ZFNs	TALENs	CRISPR/Cas9
**Origin and biological basis**	Transposable elements discovered in the 1950's in maize, specifically DNA (type II) transposons.	ϕC31 transposase-mediated integration of bacteriophage DNA into bacterial genomes	Group I introns discovered prior to 1970 in yeast that show higher than Mendelian inheritance proportions	Zinc-binding domain from transcription factors for DNA recognition, FokI endonuclease for DNA cleavage, first fused for site-specific DNA cleavage in 1996	TALE proteins discovered in *Xanthomonas* species were decoded in 2009 and conjugated to FokI endonuclease	*S. pyogenes* CRISPR/Cas9 biology for destruction and memory of parasitic nucleic acid
**Initial use for genome modification**	1982 *(D. melanogaster)*	1982 (*S. pyogenes*)	1998 (*E.coli*)	2001 (*Xenopus laevis*)	2010 (S. *cerevisiae*)	2012 (Human cell lines)
**Year used in mosquitoes**	1998	2006	2011	2013	2013	2015
**Mechanism**	Transposase mediated transfer of DNA between ITRs either from a plasmid into the mosquito genome (transformation) or from one place in a genome to another	ϕC31 integrase mediates integration of a plasmid bearing an attB site into a complementary attP site on the mosquito genome	HEG encoded endonuclease recognizes and cleaves genomic DNA such that a gene cassette can be integrated by cell HDR machinery	Pairs of zinc-finger domains recognize and bind a sequence of nucleotide triplets, each endonuclease cuts the DNA backbone, together creating a double-stranded break	Pairs of TALE domains recognize and bind a sequence of nucleotides, each endonuclease cuts DNA backbone, together creating a double-stranded break	Cas9 protein bound to a sgRNA through scaffold sequence on the sgRNA recognizes a genomic sequence that is complementary to ~20 nucleotides on the sgRNA
Mutation efficiency *	NA	NA	1–9%	<7%	<16%	>90%
Transformation efficiency *	~2%	10–18%	<1%	<6%	~2%	~2%
Gene drive *	Potentially	No	Yes	No	No	Yes
**Drive efficiency**	<<0.05%	NA	56%	NA	NA	90–100%
**Benefits**	First available transformation technique in mosquitoes, some natural transposons are extremely efficient gene drives.	Insertion into a known site	Insertion into a known site, high efficiency, recognition of sites on X-chromosome only in *An. gambiae*	Site-specific editing. Double stranded cuts allowed targeted mutagenesis-first gene editing technique for reverse genetics	Site-specific editing, efficient mutagenesis, TALEN expression plasmids could be cloned in-house.	Site-specific, easily re-engineered, adaptable to different species. Highly efficient for mutagenesis and as a drive.
**Drawbacks**	Random insertion, low transformation efficiency, very low mobility of synthetic transposon once integrated into the genome	Requires pre-insertion of the attP site using transposition	Requires pre-existing target-sites, re-engineering of the HEG, or transgenesis for insertion of target sites. Drive mechanism generates drive-resistant alleles.	Expensive, requires modular protein engineering, some codons were not recognized by any zinc-fingers, requires in vitro optimization	Requires protein engineering, timing: introduced just before Cas9 was demonstrated for gene-editing	Drive mechanism generates drive-resistant alleles
**References**	[40,42,43,44,45,46,47,48,49,50,57,173,174,175,176]	[50,132,142,143,177]	[73,75,79,152,178,179,180,181]	[144,145,146,147,154,182,183]	[148,150,153,159,184,185]	[156,159,160,161,162,163,164,166,167,169,170,171,172]

Note: * Mutation and transformation efficiency are represented slightly differently in different mosquito studies, but for a rough comparison, mutation efficiency here represents the percentage of G_1_ offspring with a deletion (not unique deletion events) and transformation efficiency is the percentage of individual surviving G_0_ that produced transgenic offspring. Gene drive efficiency is represented by the gene-conversion rate or the proportion of wild-type alleles converted to drive alleles. HEG: homing endonuclease gene; ZFN: zinc-finger nuclease; TALEN: transcription activator-like effector nuclease, ITR: inverted terminal repeat, HDR: homology-directed repair.

## References

[1] MacDonald G. (1957). The Epidemiology and Control of Malaria.

[2] Bhatt S., Weiss D., Cameron E., Bisanzio D., Mappin B. (2015). The effect of malaria control on *Plasmodium falciparum* in Africa between 2000 and 2015. Nature.

[3] Ross R. (1897). On some peculiar pigmented cells found in two mosquitos fed on malarial blood. Br. Med. J..

[4] Grassi G.B. Rapporti tra la Malaria e Peculiari Insetti (Zanzaroni e Zanzare Palustri). http://www.lincei.it/pubblicazioni/rendicontiFMN/rol/visabs.php?lang=en&type=mat&fileId=1046.

[5] Reed W., Carroll J., Agramonte A. (1901). The Etiology of the Yellow Fever. An Additional Note. J. Am. Med. Assoc..

[6] Manson P. (1878). On the development of *Filaria sanguinis hominis*, and on the mosquito considered as a nurse*. J. Linn. Soc. Lond. Zool..

[7] Kourí G., Guzmán M.G., Bravo J. (1986). Hemorrhagic dengue in Cuba: History of an epidemic. Bull. Pan Am. Health Organ..

[8] Soper F., Wilson D.B. (1943). Anopheles gambiae in Brazil, 1930 to 1940.

[9] Soper F.L. (1963). The elimination of urban yellow fever in the Americas through the eradication of Aedes aegypti. Am. J. Public Health Nations Health.

[10] Carson R. (1962). Silent Spring.

[11] NIOSH NIOSH Special Occupation Hazard Reviw: DDT. https://www.cdc.gov/niosh/docs/78-200/default.html.

[12] Muller H.J. (1927). Artificial transmutation of the gene. Science.

[13] Serebrovskii A.S. (1940). On the possibility of a new method for the control of insect pests. Zool. Zhurnal.

[14] Knipling E.F. (1955). Possibilities of insect control or eradication through the use of sexually sterile males. J. Econ. Entomol..

[15] Curtis C.F. (1985). Genetic control of insect pests: Growth industry or lead balloon?. Biol. J. Linn. Soc..

[16] Vanderplank F.L. (1944). Hybridization between *Glossina* Species and Suggested New Method for Control of Certain Species of Tsetse. Nature.

[17] Vanderplank F.L. (1947). Experiments in the hybridisation of Tsetse-flies (*Glossina*, Diptera) and the possibility of a new control method. Trans. R. Entomol. Soc. Lond..

[18] Vanderplank F.L. (1948). Experiments in Cross-Breeding Tsetse-Flies (*Glossina* Species). Ann. Trop. Med. Parasitol..

[19] WHO Scientific Group on the Genetics of Vectors and Insecticide Resistance WHO Technical Report Series No. 268; Genetics of Vectors and Insecticide Resistance. http://apps.who.int/iris/bitstream/10665/40573/1/WHO_TRS_268.pdf.

[20] Curtis C.F. (1968). Possible use of translocations to fix desirable genes in insect pest populations. Nature.

[21] Curtis C.F., Graves P.M. (1988). Methods for replacement of malaria vector populations. J. Trop. Med. Hyg..

[22] Hamilton W.D. (1967). Extraordinary Sex Ratios. Science.

[23] Knipling E.F., Laven H., Craig G.B., Pal R., Kitzmiller J.B., Smith C.N., Brown A.W. (1968). Genetic control of insects of public health importance. Bull. World Health Organ..

[24] Curtis C.F., Adak T. (1974). Population replacement in *Culex fatigans* by means of cytoplasmic incompatibility: 1. Laboratory experiments with non-overlapping generations. Bull. World Health Organ..

[25] Curtis C.F. (1976). Population replacement in *Culex fatigans* by means of cytoplasmic incompatibility: 2. Field cage experiments with overlapping generations. Bull. World Health Organ..

[26] Wood R.J., Cook L.M., Hamilton A., Whitelaw A. (1977). Transporting the Marker Gene re (Red Eye) into a Laboratory Cage Population of *Aedes Aegypti* (Diptera: Culicidae), Using Meiotic Drive at the MD Locus. J. Med. Entomol..

[27] Collins F.H., Sakai R.K., Vernick K.D., Paskewitz S., Seeley D.C., Miller L.H., Collins W.E., Campbell C.C., Gwadz R.W. (1986). Genetic selection of a *Plasmodium*-refractory strain of the malaria vector *Anopheles gambiae*. Science.

[28] Graves P.M., Curtis C.F. (1982). A cage replacement experiment involving introduction of genes for refractoriness to *Plasmodium yoelii nigeriensis* into a population of *Anopheles gambiae* (Diptera: Culicidae). J. Med. Entomol..

[29] Beaty B.J., Prager D.J., James A.A., Jacobs-Lorena M., Miller L.H., Law J.H., Collins F.H., Kafatos F.C. (2009). From Tucson to genomics and transgenics: The vector biology network and the emergence of modern vector biology. PLoS Negl. Trop. Dis..

[30] Collins F.H., James A.A. (1996). Genetic modification of mosquitoes. Sci. Med..

[31] James A.A., Blackmer K., Racioppi J.V. (1989). A salivary gland-specific, maltase-like gene of the vector *Aedes aegypti*. Gene.

[32] Meredith S.E.O., James A.A. (1990). Biotechnology as applied to vectors and vector control. Ann. Parasito. Hum. Comp..

[33] Miller L.H., Sakai R.K., Romans P., Gwadz R.W., Kantoff P., Coon H.G. (1987). Stable integration and expression of a bacterial gene in the mosquito *Anopheles gambiae*. Science.

[34] Burt A., Trivers R. (2006). Genes in Conflict: The Biology of Selfish Genetic Elements.

[35] McClintock B. (1987). The Discovery and Characterization of Tranposable Elements: The Collected Papers of Barbara McClintock.

[36] Engels W.R. (1997). Invasions of P elements. Genetics.

[37] Charlesworth B., Sniegowski P., Stephan W. (1994). The evolutionary dynamics of repetitive DNA in eukaryotes. Nature.

[38] Mackay T. (1986). Transposable element-induced fitness mutations in *Drosophil*a *melanogaster*. Genet. Res..

[39] Fitzpatrick B., Sved J. (1986). High levels of fitness modifiers induced by hybrid dysgenesis in *Drosophila melanogaster*. Genet. Res..

[40] Spradling A.C., Rubin G.M. (1982). Transposition of cloned P elements into *Drosophila* germ line chromosomes. Science.

[41] Warren A., Crampton J., Hagedorn H., Hildebrand H., Kidwell M., Law J. (1990). Transposable genetic elements in the genome of the mosquito, *Aedes aegypti*. Molecular Insect Science.

[42] Allen M.L., O’Brochta D.A., Atkinson P.W., Levesque C.S. (2001). Stable, Germ-line Transformation of *Culex quinquefasciatus* (Diptera: Culicidae). J. Med. Entomol..

[43] Grossman G.L., Rafferty C.S., Clayton J.R., Stevens T.K., Mukabayire O., Benedict M.Q. (2001). Germline transformation of the malaria vector, *Anopheles gambiae*, with the *piggyBac* transposable element. Insect Mol. Biol..

[44] Rodrigues F., Oliveira S., Rocha B. (2006). Germline transformation of *Aedes fluviatilis* (Diptera: Culicidae) with the *piggyBac* transposable element. Mem. Inst. Oswaldo Cruz.

[45] Nolan T., Bower T., Brown A., Crisanti A. (2002). *piggyBac*-mediated germline transformation of the malaria mosquito *Anopheles stephensi* using the red fluorescent protein DsRed as a selectable marker. J. Biol..

[46] Perera O., Harrell I., Handler A. (2002). Germ-line transformation of the South American malaria vector, *Anopheles albimanus*, with a *piggyBac*/EGFP transposon vector is routine and highly efficient. Insect Mol. Biol..

[47] Catteruccia F., Nolan T., Loukeris T.G., Blass C., Savakis C., Kafatos F.C., Crisanti A. (2000). Stable germline transformation of the malaria mosquito *Anopheles stephensi*. Nature.

[48] Jasinskiene N., Coates C.J., Benedict M.Q., Cornel A.J., Rafferty C.S., James A.A., Collins F.H. (1998). Stable transformation of the yellow fever mosquito, *Aedes aegypti*, with the Hermes element from the housefly. Proc. Natl. Acad. Sci. USA.

[49] Coates C.J., Jasinskiene N., Miyashiro L., James A.A. (1998). Mariner transposition and transformation of the yellow fever mosquito, *Aedes aegypti*. Proc. Natl. Acad. Sci. USA.

[50] Labbé G.M.C., Nimmo D.D., Alphey L. (2010). *piggybac*- and PhiC31-mediated genetic transformation of the Asian tiger mosquito, *Aedes albopictus* (Skuse). PLoS Negl. Trop. Dis..

[51] Adelman Z.N., Basu S., Myles K.M., Adelman Z.N. (2016). Gene insertions and deletion in mosquitoes. Genetic Control of Malaria and Dengue.

[52] Kidwell M., Ribeiro J. (1992). Can transposable elements be used to drive disease refractoriness genes into vector populations?. Parasitol. Today.

[53] James A.A. (2005). Gene drive systems in mosquitoes: Rules of the road. Trends Parasitol..

[54] Gould F., Schliekelman P. (2004). Population genetics of autocidal control and strain replacement. Annu. Rev. Entomol..

[55] Rasgon J.L., Gould F. (2005). Transposable element insertion location bias and the dynamics of gene drive in mosquito populations. Insect Mol. Biol..

[56] Scali C., Nolan T., Sharakhov I., Sharakhova M., Crisanti A., Catteruccia F. (2007). Post-integration behavior of a Minos transposon in the malaria mosquito *Anopheles stephensi*. Mol. Genet. Genom..

[57] O’Brochta D.A., Sethuraman N., Wilson R., Hice R.H., Pinkerton A.C., Levesque C.S., Bideshi D.K., Jasinskiene N., Coates C.J., James A.A. (2003). Gene vector and transposable element behavior in mosquitoes. J. Exp. Biol..

[58] Macias V.M., Jimenez A.J., Burini-Kojin B., Pledger D., Jasinskiene N., Phong C.H., Chu K., Fazekas A., Martin K., Marinotti O. (2017). *nanos*-Driven expression of *piggyBac* transposase induces mobilization of a synthetic autonomic transposon in *Anopheles stephensi*. Insect Biochem. Mol. Biol..

[59] O’Brochta D.A., Alford R.T., Pilitt K.L., Aluvihare C.U., Harrell R.A. (2011). *piggyBac* transposon remobilization and enhancer detection in *Anopheles mosquitoes*. Proc. Natl. Acad. Sci. USA.

[60] Davis S., Bax N., Grewe P. (2001). Engineered underdominance allows efficient and economical introgression of traits into pest populations. J. Theor. Biol..

[61] Akbari O.S., Matzen K.D., Marshall J.M., Huang H., Ward C.M., Hay B.A. (2013). A Synthetic Gene Drive System for Local, Reversible Modification and Suppression of Insect Populations. Curr. Biol..

[62] Yen J.H., Barr A.R. (1973). The etiological agent of cytoplasmic incompatibility in *Culex pipiens*. J. Invertebr. Pathol..

[63] Yen J.H., Barr A.R. (1971). New hypothesis of the cause of cytoplasmic incompatibility in *Culex pipiens* L.. Nature.

[64] Stouthamer R., Breeuwer J.A.J., Hurst G.D.D. (1999). *Wolbachia pipientis*: Microbial Manipulator of Arthropod Reproduction. Annu. Rev. Microbiol..

[65] LePage D.P., Metcalf J.A., Bordenstein S.R., On J., Perlmutter J.I., Shropshire J.D., Layton E.M., Funkhouser-Jones L.J., Beckmann J.F., Bordenstein S.R. (2017). Prophage WO genes recapitulate and enhance *Wolbachia*-induced cytoplasmic incompatibility. Nature.

[66] Sinkins S., Curtis C., O’Neill S., O’Neill S.L., Hoffman A.A., Werren J.H. (1997). The potential application of inherited symbiont systems to pest control. Explore our Research. Influential Passangers: Inherited Microorganisms and Arthropod Reproduction.

[67] Turelli M., Hoffmann A.A. (1999). Microbe-induced cytoplasmic incompatibility as a mechanism for introducing transgenes into arthropod populations. Insect Mol. Biol..

[68] Wade M.J., Beeman R.W. (1994). The population dynamics of maternal-effect selfish genes. Genetics.

[69] Beeman R., Friesen K., Denell R. (1992). Maternal-effect selfish genes in flour beetles. Science.

[70] Chen C., Huang H., Ward C.M., Su J.T., Schaeffer L.V., Guo M., Hay B.A. (2007). A Synthetic Maternal-Effect Selfish Genetic Element Drives Population Replacement in *Drosophila*. Science.

[71] Belfort M., Derbyshire V., Parker M.M., Cousineau B., Lambowitz A.M. (2002). Mobile Introns: Pathways and Proteins. Mobile DNA II.

[72] Chevalier B.S., Stoddard B.L. (2001). Homing endonucleases: Structural and functional insight into the catalysts of intron/intein mobility. Nucleic Acids Res..

[73] Coen D., Deutsch J., Netter P., Petrochilo E. (1970). Mitochondrial genetics. I. Methodology and phenomenology. Symp. Soc. Exp. Biol..

[74] Stoddard B.L. (2005). Homing endonuclease structure and function. Q. Rev. Biophys..

[75] Burt A. (2003). Site-specific selfish genes as tools for the control and genetic engineering of natural populations. Proc. Biol. Sci..

[76] Ashworth J., Taylor G., Havranek J. (2010). Computational reprogramming of homing endonuclease specificity at multiple adjacent base pairs. Nucleic Acids Res..

[77] Ashworth J., Havranek J., Duarte C., Sussman D. (2006). Computational redesign of endonuclease DNA binding and cleavage specificity. Nature.

[78] Jarjour J., West-Foyle H., Certo M. (2009). High-resolution profiling of homing endonuclease binding and catalytic specificity using yeast surface display. Nucleic Acids Res..

[79] Windbichler N., Miriam M., Papathanos P.A., Thyme S.B., Li H., Ulge U.Y., Hovde B.T., Baker D., Monnat R.J., College B. (2011). A synthetic homing endonuclease-based gene drive system in the human malaria mosquito. Nature.

[80] Laven H. (1967). Eradication of *Culex pipiens fatigans* through Cytoplasmic Incompatibility. Nature.

[81] Kriesner P., Hoffmann A.A., Lee S.F., Turelli M., Weeks A.R. (2013). Rapid Sequential Spread of Two *Wolbachia* Variants in *Drosophila simulans*. PLoS Pathog..

[82] Collins F.H., Paskewitz S.M. (1995). Malaria: Current and Future Prospects for Control. Annu. Rev. Entomol..

[83] Sinkins S.P., O’Neill S.L. (2000). *Wolbachia* as a vehicle to modify insect populations. Insect Transgenesis: Methods and Applications.

[84] Calvitti M., Moretti R., Lampazzi E., Bellini R., Dobson S.L. (2010). Characterization of a new *Aedes albopictus* (Diptera: Culicidae)-*Wolbachia pipientis* (Rickettsiales: Rickettsiaceae) symbiotic association generated by artificial transfer of the *w*Pip strain from *Culex pipiens* (Diptera: Culicidae). J. Med. Entomol..

[85] Calvitti M., Moretti R., Skidmore A.R., Dobson S.L. (2012). *Wolbachia* strain *w*Pip yields a pattern of cytoplasmic incompatibility enhancing a *Wolbachia*-based suppression strategy against the disease vector *Aedes albopictus*. Parasit. Vectors.

[86] Moretti R., Calvitti M. (2013). Male mating performance and cytoplasmic incompatibility in a *w*Pip *Wolbachia* trans-infected line of *Aedes albopictus* (*Stegomyia albopicta*). Med. Vet. Entomol..

[87] Puggioli A., Calvitti M., Moretti R., Bellini R. (2016). *w*Pip *Wolbachia* contribution to *Aedes albopictus* SIT performance: Advantages under intensive rearing. Acta Trop..

[88] MosquitoMate. http://mosquitomate.com/?v=3.0.

[89] Walker T., Johnson P.H., Moreira L.A., Iturbe-Ormaetxe I., Frentiu F.D., McMeniman C.J., Leong Y.S., Dong Y., Axford J., Kriesner P. (2011). The *w*Mel Wolbachia strain blocks dengue and invades caged *Aedes aegypti* populations. Nature.

[90] Hoffmann A.A., Montgomery B.L., Popovici J., Iturbe-Ormaetxe I., Johnson P.H., Muzzi F., Greenfield M., Durkan M., Leong Y.S., Dong Y. (2011). Successful establishment of Wolbachia in *Aedes* populations to suppress dengue transmission. Nature.

[91] Frentiu F.D., Zakir T., Walker T., Popovici J., Pyke A.T., van den Hurk A., McGraw E.A., O’Neill S.L. (2014). Limited Dengue Virus Replication in Field-Collected *Aedes aegypti* Mosquitoes Infected with *Wolbachia*. PLoS Negl. Trop. Dis..

[92] Ye Y.H., Carrasco A.M., Frentiu F.D., Chenoweth S.F., Beebe N.W., van den Hurk A.F., Simmons C.P., O’Neill S.L., McGraw E.A. (2015). *Wolbachia* Reduces the Transmission Potential of Dengue-Infected *Aedes aegypti*. PLoS Negl. Trop. Dis..

[93] Weinert L.A., Araujo-Jnr E.V., Ahmed M.Z., Welch J.J. (2015). The incidence of bacterial endosymbionts in terrestrial arthropods. Proc. R. Soc. B Biol. Sci..

[94] McNaughton D. (2012). The Importance of Long-Term Social Research in Enabling Participation and Developing Engagement Strategies for New Dengue Control Technologies. PLoS Negl. Trop. Dis..

[95] Nikoh N., Tanaka K., Shibata F., Kondo N., Hizume M., Shimada M., Fukatsu T. (2008). *Wolbachia* genome integrated in an insect chromosome: Evolution and fate of laterally transferred endosymbiont genes. Genome Res..

[96] Wu M., Sun L.V., Vamathevan J., Riegler M., Deboy R., Brownlie J.C., McGraw E.A., Martin W., Esser C., Ahmadinejad N. (2004). Phylogenomics of the reproductive parasite *Wolbachia pipientis*
*w*Mel: A streamlined genome overrun by mobile genetic elements. PLoS Biol..

[97] Kondo N., Nikoh N., Ijichi N. (2002). Genome fragment of *Wolbachia* endosymbiont transferred to X chromosome of host insect. Proc. Natl. Acad. Sci. USA.

[98] Klasson L., Kambris Z., Cook P. (2009). Horizontal gene transfer between *Wolbachia* and the mosquito *Aedes aegypti*. BMC. Genom..

[99] Fenn K., Conlon C., Jones M., Quail M. (2006). Phylogenetic relationships of the *Wolbachia* of nematodes and arthropods. PLoS Pathog..

[100] Hotopp J.C.D. (2011). Horizontal gene transfer between bacteria and animals. Trends Genet..

[101] Hotopp J.C.D., Clark M.E., Oliveira D.C.S.G., Foster J.M., Fischer P., Torres M.C.M., Giebel J.D., Kumar N., Ishmael N., Wang S. (2007). Widespread Lateral Gene Transfer from Intracellular Bacteria to Multicellular Eukaryotes. Science.

[102] Foster J., Ganatra M., Kamal I., Ware J., Makarova K. (2005). The *Wolbachia* genome of *Brugia malayi*: Endosymbiont evolution within a human pathogenic nematode. PLoS Biol..

[103] Fu G., Lees R.S., Nimmo D., Aw D., Jin L., Gray P., Berendonk T.U., White-Cooper H., Scaife S., Kim Phuc H. (2010). Female-specific flightless phenotype for mosquito control. Proc. Natl. Acad. Sci. USA.

[104] Atkinson M.P., Su Z., Alphey N., Alphey L.S., Coleman P.G., Wein L.M. (2007). Analyzing the control of mosquito-borne diseases by a dominant lethal genetic system. Proc. Natl. Acad. Sci. USA.

[105] Phuc H., Andreasen M.H., Burton R.S., Vass C., Epton M.J., Pape G., Fu G., Condon K.C., Scaife S., Donnelly C.A. (2007). Late-acting dominant lethal genetic systems and mosquito control. BMC Biol..

[106] Thomas D.D., Donnelly C.A., Wood R.J., Alphey L.S. (2000). Insect population control using a dominant, repressible, lethal genetic system. Science.

[107] Carvalho D.O., McKemey A.R., Garziera L., Lacroix R., Donnelly C.A., Alphey L., Malavasi A., Capurro M.L. (2015). Suppression of a Field Population of *Aedes aegypti* in Brazil by Sustained Release of Transgenic Male Mosquitoes. PLoS Negl. Trop. Dis..

[108] Harris A.F., McKemey A.R., Nimmo D., Curtis Z., Black I., Morgan S.A., Oviedo M.N., Lacroix R., Naish N., Morrison N.I. (2012). Successful suppression of a field mosquito population by sustained release of engineered male mosquitoes. Nat. Biotechnol..

[109] Labbé G.M.C., Scaife S., Morgan S.A., Curtis Z.H., Alphey L. (2012). Female-Specific Flightless (fsRIDL) Phenotype for Control of *Aedes albopictus*. PLoS Negl. Trop. Dis..

[110] Marinotti O., Jasinskiene N., Fazekas A., Scaife S., Fu G., Mattingly S.T., Chow K., Brown D.M., Alphey L., James A.A. (2013). Development of a population suppression strain of the human malaria vector mosquito, *Anopheles stephensi*. Malar. J..

[111] Lin H., McGrath J., Wang P., Lee T. (2006). Cellular Toxicity Induced by SRF-Mediated Transcriptional Squelching. Toxicol. Sci..

[112] Environmental Assessment for Investigational Use of *Aedes aegypti* OX513A. https://www.fda.gov/downloads/AnimalVeterinary/DevelopmentApprovalProcess/GeneticEngineering/GeneticallyEngineeredAnimals/UCM514698.pdf.

[113] Clyde K., Kyle J.L., Harris E. (2006). Recent advances in deciphering viral and host determinants of dengue virus replication and pathogenesis. J. Virol..

[114] Dong Y., Das S., Cirimotich C., Souza-Neto J.A., McLean K.J., Dimopoulos G. (2011). Engineered anopheles immunity to *Plasmodium* infection. PLoS Pathog..

[115] Erickson S.M., Xi Z., Mayhew G.F., Ramirez J.L., Aliota M.T., Christensen B.M., Dimopoulos G. (2009). Mosquito infection responses to developing filarial worms. PLoS Negl. Trop. Dis..

[116] Ghosh A.K., Coppens I., Gårdsvoll H., Ploug M., Jacobs-Lorena M. (2011). *Plasmodium* ookinetes coopt mammalian plasminogen to invade the mosquito midgut. Proc. Natl. Acad. Sci. USA.

[117] Kang S., Shields A.R., Jupatanakul N., Dimopoulos G., Mongin E. (2014). Suppressing Dengue-2 Infection by Chemical Inhibition of *Aedes aegypti* Host Factors. PLoS Negl. Trop. Dis..

[118] Kuadkitkan A., Wikan N., Fongsaran C., Smith D.R. (2010). Identification and characterization of prohibitin as a receptor protein mediating DENV-2 entry into insect cells. Virology.

[119] Olson K.E., Adelman Z.N., Travanty E.A., Sanchez-Vargas I., Beaty B.J., Blair C.D. (2002). Developing arbovirus resistance in mosquitoes. Insect Biochem. Mol. Biol..

[120] Osta M.A., Christophides G.K., Vlachou D., Kafatos F.C. (2004). Innate immunity in the malaria vector *Anopheles gambiae*: Comparative and functional genomics. J. Exp. Biol..

[121] Pike A., Vadlamani A., Sandiford S.L., Gacita A., Dimopoulos G. (2014). Characterization of the Rel2-regulated transcriptome and proteome of *Anopheles stephensi* identifies new anti-*Plasmodium* factors. Insect Biochem. Mol. Biol..

[122] Niare O., Markianos K., Volz J., Oduol F., Touré A., Bagayoko M., Sangaré D., Traoré S.F., Wang R., Blass C. (2002). Genetic Loci Affecting Resistance to Human Malaria Parasites in a West African Mosquito Vector Population. Science.

[123] Riehle M.M., Markianos K., Lambrechts L., Xia A., Sharakhov I., Koella J.C., Vernick K.D. (2007). A major genetic locus controlling natural *Plasmodium falciparum* infection is shared by East and West African *Anopheles gambiae*. Malar. J..

[124] Riehle M.A., Moreira C.K., Lampe D., Lauzon C., Jacobs-Lorena M. (2007). Using bacteria to express and display anti-*Plasmodium* molecules in the mosquito midgut. Int. J. Parasitol..

[125] Ramirez J.L., Dimopoulos G. (2010). The Toll immune signaling pathway control conserved anti-dengue defenses across diverse *Ae. aegypti* strains and against multiple dengue virus serotypes. Dev. Comp. Immunol..

[126] Smith R.C., Barillas-Mury C., Jacobs-Lorena M. (2015). Hemocyte differentiation mediates the mosquito late-phase immune response against *Plasmodium* in *Anopheles gambiae*. Proc. Natl. Acad. Sci. USA.

[127] Corby-Harris V., Drexler A., Watkins de Jong L., Antonova Y., Pakpour N., Ziegler R., Ramberg F., Lewis E.E., Brown J.M., Luckhart S. (2010). Activation of *Akt* signaling reduces the prevalence and intensity of malaria parasite infection and lifespan in *Anopheles stephensi* mosquitoes. PLoS Pathog..

[128] Hauck E.S., Antonova-Koch Y., Drexler A., Pietri J., Pakpour N., Liu D., Blacutt J., Riehle M.A., Luckhart S. (2013). Overexpression of phosphatase and tensin homolog improves fitness and decreases *Plasmodium falciparum* development in *Anopheles stephensi*. Microbes Infect..

[129] Luckhart S., Giulivi C., Drexler A.L., Antonova-Koch Y., Sakaguchi D., Napoli E., Wong S., Price M.S., Eigenheer R., Phinney B.S. (2013). Sustained activation of Akt elicits mitochondrial dysfunction to block *Plasmodium falciparum* infection in the mosquito host. PLoS Pathog..

[130] Ito J., Ghosh A., Moreira L.A., Wimmer E.A., Jacobs-Lorena M. (2002). Transgenic anopheline mosquitoes impaired in transmission of a malaria parasite. Nature.

[131] Adelman Z.N., Basu S., Myles K.M., Adelman Z.N. (2016). Engineering pathogen resistance in mosquitoes. Genetic Control of Malaria and Dengue.

[132] Isaacs A.T., Jasinskiene N., Tretiakov M., Thiery I., Zettor A., Bourgouin C., James A.A. (2012). Transgenic *Anopheles stephensi* coexpressing single-chain antibodies resist *Plasmodium falciparum* development. Proc. Natl. Acad. Sci. USA.

[133] Mathur G., Sanchez-Vargas I., Alvarez D., Olson K.E., Marinotti O., James A.A. (2010). Transgene-mediated suppression of dengue viruses in the salivary glands of the yellow fever mosquito, *Aedes aegypti*. Insect Mol. Biol..

[134] Fraser M.J., Ciszczon T., Elick T., Bauser C. (1996). Precise excision of TTAA-specific lepidopteran transposons *piggyBac* (IFP2) and tagalong (TFP3) from the baculovirus genome in cell lines from two species of Lepidoptera. Insect Mol. Biol..

[135] Arcà B., Zabalou S., Loukeris T., Savakis C. (1997). Mobilization of a Minos transposon in *Drosophila melanogaster* chromosomes and chromatid repair by heteroduplex formation. Genetics.

[136] Spradling A., Stern D., Beaton A., Rhem E. (1999). The Berkeley *Drosophila* Genome Project gene disruption project: Single P-element insertions mutating 25% of vital *Drosophila* genes. Genetics.

[137] Spradling A., Stern D., Kiss I. (1995). Gene disruptions using P transposable elements: An integral component of the *Drosophila* genome project. Proc. Natl. Acad. Sci. USA.

[138] Lyman R., Lawrence F., Nuzhdin S., Mackay T. (1996). Effects of single P-element insertions on bristle number and viability in *Drosophila melanogaster*. Genetics.

[139] Marrelli M.T., Moreira C.K., Kelly D., Alphey L., Jacobs-Lorena M. (2006). Mosquito transgenesis: What is the fitness cost?. Trends Parasitol..

[140] Takeuchi R., Choi M., Stoddard B.L. (2014). Redesign of extensive protein-DNA interfaces of meganucleases using iterative cycles of in vitro compartmentalization. Proc. Natl. Acad. Sci. USA.

[141] Windbichler N., Papathanos P.A., Catteruccia F., Ranson H., Burt A., Crisanti A. (2007). Homing endonuclease mediated gene targeting in *Anopheles gambiae* cells and embryos. Nucleic Acids Res..

[142] Nimmo D.D., Alphey L., Meredith J.M., Eggleston P. (2006). High efficiency site-specific genetic engineering of the mosquito genome. Insect Mol. Biol..

[143] Meredith J.M., Basu S., Nimmo D.D., Larget-Thiery I., Warr E.L., Underhill A., McArthur C.C., Carter V., Hurd H., Bourgouin C. (2011). Site-specific integration and expression of an anti-malarial gene in transgenic *Anopheles gambiae* significantly reduces *Plasmodium* infections. PLoS ONE.

[144] Beerli R.R., Barbas C.F. (2002). Engineering polydactyl zinc-finger transcription factors. Nat. Biotechnol..

[145] Liu Q., Segal D.J., Ghiara J.B., Barbas C.F. (1997). Design of polydactyl zinc-finger proteins for unique addressing within complex genomes. Proc. Natl. Acad. Sci. USA.

[146] Miller J.C., Holmes M.C., Wang J., Guschin D.Y., Lee Y.-L., Rupniewski I., Beausejour C.M., Waite A.J., Wang N.S., Kim K.A. (2007). An improved zinc-finger nuclease architecture for highly specific genome editing. Nat. Biotechnol..

[147] Kim Y.-G., Cha J., Chandrasegaran S. (1996). Hybrid restriction enzymes: Zinc finger fusions to Fok I cleavage domain (Flavobacterium okeanokoites/chimeric restriction endonuclease/protein engineering/recognition and cleavage domains). Proc. Natl. Acad. Sci. USA.

[148] Christian M., Cermak T., Doyle E.L., Schmidt C., Zhang F., Hummel A., Bogdanove A.J., Voytas D.F. (2010). Targeting DNA Double-Strand Breaks with TAL Effector Nucleases. Genetics.

[149] Boch J., Scholze H., Schornack S., Landgraf A., Hahn S., Kay S., Lahaye T., Nickstadt A., Bonas U. (2009). Breaking the code of DNA binding specificity of TAL-type III effectors. Science.

[150] Moscou M.J., Bogdanove A.J. (2009). A simple cipher governs DNA recognition by TAL effectors. Science.

[151] Kim Y., Kweon J., Kim A., Chon J.K., Yoo J.Y., Kim H.J., Kim S., Lee C., Jeong E., Chung E. (2013). A library of TAL effector nucleases spanning the human genome. Nat. Biotechnol..

[152] Aryan A., Anderson M.A.E., Myles K.M., Adelman Z.N. (2013). Germline excision of transgenes in *Aedes aegypti* by homing endonucleases. Sci. Rep..

[153] Smidler A.L., Terenzi O., Soichot J., Levashina E.A., Marois E. (2013). Targeted mutagenesis in the malaria mosquito using TALE nucleases. PLoS ONE.

[154] DeGennaro M., McBride C.S., Seeholzer L., Nakagawa T., Dennis E.J., Goldman C., Jasinskiene N., James A.A., Vosshall L.B. (2013). *orco* mutant mosquitoes lose strong preference for humans and are not repelled by volatile DEET. Nature.

[155] Guo H., Karberg M., Long M., Jones J.P., Sullenger B., Lambowitz A.M. (2000). Group II Introns Designed to Insert into Therapeutically Relevant DNA Target Sites in Human Cells. Science.

[156] Jinek M., Chylinski K., Fonfara I., Hauer M., Doudna J.A., Charpentier E. (2012). A programmable dual-RNA-guided DNA endonuclease in adaptive bacterial immunity. Science.

[157] Mali P., Yang L., Esvelt K.M., Aach J., Guell M., DiCarlo J.E., Norville J.E., Church G.M. (2013). RNA-guided human genome engineering via Cas9. Science.

[158] Cong L., Ran F.A., Cox D., Lin S., Barretto R., Habib N., Hsu P.D., Wu X., Jiang W., Marraffini L.A. (2013). Multiplex Genome Engineering Using CRISPR/Cas Systems. Science.

[159] Basu S., Aryan A., Overcash J.M., Samuel G.H., Anderson M.A.E., Dahlem T.J., Myles K.M., Adelman Z.N. (2015). Silencing of end-joining repair for efficient site-specific gene insertion after TALEN/CRISPR mutagenesis in *Aedes aegypti*. Proc. Natl. Acad. Sci. USA.

[160] Dong S., Lin J., Held N.L., Clem R.J., Passarelli A.L., Franz A.W.E. (2015). Heritable CRISPR/Cas9-Mediated Genome Editing in the Yellow Fever Mosquito, *Aedes aegypti*. PLoS ONE.

[161] Kistler K.E., Vosshall L.B., Matthews B.J. (2015). Genome Engineering with CRISPR-Cas9 in the Mosquito *Aedes aegypti*. Cell Rep..

[162] Hall A.B., Basu S., Jiang X., Qi Y., Timoshevskiy V.A., Biedler J.K., Sharakhova M.V., Elahi R., Anderson M.A.E., Chen X.-G. (2015). A male-determining factor in the mosquito *Aedes aegypti*. Science.

[163] Hammond A., Galizi R., Kyrou K., Simoni A., Siniscalchi C., Katsanos D., Gribble M., Baker D., Marois E., Russell S. (2015). A CRISPR-Cas9 gene drive system targeting female reproduction in the malaria mosquito vector *Anopheles gambiae*. Nat. Biotechnol..

[164] Gantz V.M., Jasinskiene N., Tatarenkova O., Fazekas A., Macias V.M., Bier E., James A.A. (2015). Highly efficient Cas9-mediated gene drive for population modification of the malaria vector mosquito *Anopheles stephensi*. Proc. Natl. Acad. Sci. USA.

[165] Gantz V.M., Bier E. (2015). The mutagenic chain reaction: A method for converting heterozygous to homozygous mutations. Science..

[166] Li M., Akbari O.S., White B.J. (2017). Highly efficient site-specific mutagenesis in malaria mosquitoes using CRISPR. bioRxiv.

[167] Deveau H., Garneau J.E., Moineau S. (2010). CRISPR/Cas System and Its Role in Phage-Bacteria Interactions. Annu. Rev. Microbiol..

[168] Wiedenheft B., Sternberg S.H., Doudna J.A. (2012). RNA-guided genetic silencing systems in bacteria and archaea. Nature.

[169] Ishino Y., Shinagawa H., Makino K., Amemura M., Nakata A. (1987). Nucleotide sequence of the *iap* gene, responsible for alkaline phosphatase isozyme conversion in *Escherichia coli*, and identification of the gene product. J. Bacteriol..

[170] Gasiunas G., Barrangou R., Horvath P., Siksnys V. (2012). Cas9-crRNA ribonucleoprotein complex mediates specific DNA cleavage for adaptive immunity in bacteria. Proc. Natl. Acad. Sci. USA.

[171] Brouns S.J.J., Jore M.M., Lundgren M., Westra E.R., Slijkhuis R.J.H., Snijders A.P.L., Dickman M.J., Makarova K.S., Koonin E.V., van der Oost J. (2008). Small CRISPR RNAs Guide Antiviral Defense in Prokaryotes. Science.

[172] Li M., Bui M., Yang T., White B.J., Akbari O.S. (2017). Germline Cas9 Expression Yields Highly Efficient Genome Engineering in a Major Worldwide Disease Vector. Aedes Aegypti. bioRxiv.

[173] Smith R.C., Atkinson P.W. (2011). Mobility properties of the Hermes transposable element in transgenic lines of *Aedes aegypti*. Genetica.

[174] Wilson R., Orsetti J., Klocko A.D., Aluvihare C., Peckham E., Atkinson P.W., Lehane M.J., O’Brochta D.A. (2003). Post-integration behavior of a *Mos1* mariner gene vector in *Aedes aegypti*. Insect Biochem. Mol. Biol..

[175] Arensburger P., Kim Y.-J., Orsetti J., Aluvihare C., O’Brochta D.A., Atkinson P.W. (2005). An active transposable element, *Herves*, from the African malaria mosquito *Anopheles gambiae*. Genetics.

[176] Sethuraman N., Fraser M.J., Eggleston P., O’Brochta D.A. (2007). Post-integration stability of *piggyBac* in *Aedes aegypti*. Insect Biochem. Mol. Biol..

[177] Rodicio M.R., Chater K.F. (1982). Small DNA-free liposomes stimulate transfection of streptomyces protoplasts. J. Bacteriol..

[178] Sellem C.H., Belcour L. (1997). Intron open reading frames as mobile elements and evolution of a group I intron. Mol. Biol. Evol..

[179] Galizi R., Doyle L.A., Menichelli M., Bernardini F., Deredec A., Burt A., Stoddard B.L., Windbichler N., Crisanti A. (2014). A synthetic sex ratio distortion system for the control of the human malaria mosquito. Nat. Commun..

[180] Roman J., Woodson S.A. (1998). Integration of the *Tetrahymen*a group I intron into bacterial rRNA by reverse splicing in vivo. Proc. Natl. Acad. Sci. USA.

[181] Bernardini F., Galizi R., Menichelli M., Papathanos P.-A., Dritsou V., Marois E., Crisanti A., Windbichler N. (2014). Site-specific genetic engineering of the *Anopheles gambiae* Y chromosome. Proc. Natl. Acad. Sci. USA.

[182] McMeniman C., Corfas R., Matthews B., Ritchie S. (2014). Multimodal integration of carbon dioxide and other sensory cues drives mosquito attraction to humans. Cell.

[183] Liesch J., Bellani L.L., Vosshall L.B., Riehle M., Brown M. (2013). Functional and Genetic Characterization of Neuropeptide Y-Like Receptors in *Aedes aegypti*. PLoS Negl. Trop. Dis..

[184] Bibikova M., Beumer K., Trautman J.K., Carroll D. (2003). Enhancing gene targeting with designed zinc finger nucleases. Science.

[185] Aryan A., Anderson M.A.E., Myles K.M., Adelman Z.N. (2013). TALEN-based gene disruption in the dengue vector *Aedes aegypti*. PLoS ONE.

[186] Kohno H., Suenami S., Takeuchi H., Sasaki T., Kubo T. (2016). Production of Knockout Mutants by CRISPR/Cas9 in the European Honeybee, *Apis mellifera* L.. Zool. Sci..

[187] Chen L., Wang G., Zhu Y.-N., Xiang H., Wang W. (2016). Advances and perspectives in the application of CRISPR/Cas9 in insects. Zool. Res..

[188] Chang H., Liu Y., Ai D., Jiang X., Dong S., Wang G. (2017). A Pheromone Antagonist Regulates Optimal Mating Time in the Moth *Helicoverpa armigera*. Curr. Biol..

[189] Yu Z., Ren M., Wang Z., Zhang B., Rong Y.S., Jiao R., Gao G. (2013). Highly Efficient Genome Modifications Mediated by CRISPR/Cas9 in *Drosophila*. Genetics.

[190] Ishizu H., Iwasaki Y.W., Hirakata S., Ozaki H., Iwasaki W., Siomi H., Siomi M.C. (2015). Somatic Primary piRNA Biogenesis Driven by *cis*-Acting RNA Elements and *trans*-Acting Yb. Cell Rep..

[191] Gratz S.J., Ukken F.P., Rubinstein C.D., Thiede G., Donohue L.K., Cummings A.M., O’Connor-Giles K.M. (2014). Highly specific and efficient CRISPR/Cas9-catalyzed homology-directed repair in *Drosophila*. Genetics.

[192] Bassett A.R., Tibbit C., Ponting C.P., Liu J.-L. (2013). Highly efficient targeted mutagenesis of *Drosophila* with the CRISPR/Cas9 system. Cell Rep..

[193] Sebo Z.L., Lee H.B., Peng Y., Guo Y. (2014). A simplified and efficient germline-specific CRISPR/Cas9 system for *Drosophila* genomic engineering. Fly.

[194] Awata H., Watanabe T., Hamanaka Y., Mito T., Noji S., Mizunami M. (2015). Knockout crickets for the study of learning and memory: Dopamine receptor Dop1 mediates aversive but not appetitive reinforcement in crickets. Sci. Rep..

[195] Li Y., Zhang J., Chen D., Yang P., Jiang F., Wang X., Kang L. (2016). CRISPR/Cas9 in locusts: Successful establishment of an olfactory deficiency line by targeting the mutagenesis of an odorant receptor co-receptor (Orco). Insect Biochem. Mol. Biol..

[196] Heinze S.D., Kohlbrenner T., Ippolito D., Meccariello A., Burger A., Mosimann C., Saccone G., Bopp D. (2017). CRISPR-Cas9 targeted disruption of the *yellow* ortholog in the housefly identifies the *brown body* locus. Sci. Rep..

[197] Gilles A.F., Schinko J.B., Averof M. (2015). Efficient CRISPR-mediated gene targeting and transgene replacement in the beetle *Tribolium castaneum*. Development.

[198] Afrane Y.A., Bonizzoni M., Yan G. (2016). Secondary Malaria Vectors of Sub-Saharan Africa: Threat to Malaria Elimination on the Continent?. Curr. Top. Malar..

[199] Alphey L., Beard C.B., Billingsley P., Coetzee M., Crisanti A., Curtis C., Eggleston P., Godfray C., Hemingway J., Jacobs-Lorena M. (2002). Malaria control with genetically manipulated insect vectors. Science.

[200] Harbach R.E. (2013). The Phylogeny and Classification of Anopheles. Anopheles Mosquitoes—New Insights into Malaria Vectors.

[201] Service M.W., Townson H. (2002). The Anopheles vector. Essent. Malariol..

[202] Patterson R.S., Weidhaas D.E., Ford H.R., Lofgren C.S. (1970). Suppression and Elimination of an Island Population of *Culex pipiens* quinquefasciatus with Sterile Males. Science.

[203] Asman S.M., McDonald P.T., Prout T. (1981). Field Studies of Genetic Control Systems for Mosquitoes. Annu. Rev. Entomol..

[204] Weidhaas D., Schmidt C., Seabrook E. (1962). Field studies on the release of sterile males for the control of Anopheles quadrimaculatus. Mosq. News.

[205] Morlan H.B., McCray E.M., Kilpatrick J.W. (1962). Field Tests with Sexually Sterile Males for control of *Aedes aegypt*i. Mosq. News.

[206] Krishnamurthy B.S., Ray S.N., Joshi G. (1962). A note on preliminary field studies of the use of irradiated males for reduction of *C. fatigans* Wied populations. Indian J. Malariol..

[207] Spielman A. (1994). Why entomological antimalaria research should not focus on transgenic mosquitoes. Parasitol. Today.

[208] Benedict M.Q., Robinson A.S. (2003). The first releases of transgenic mosquitoes: An argument for the sterile insect technique. Trends Parasitol..

[209] Lambrechts L., Koella J.C., Boëte C. (2008). Can transgenic mosquitoes afford the fitness cost?. Trends Parasitol..

[210] Alphey L., Benedict M., Bellini R., Clark G.G., Dame D.A., Service M.W., Dobson S.L. (2010). Sterile-insect methods for control of mosquito-borne diseases: An analysis. Vector Borne Zoonotic Dis..

[211] Fitz-Earle M., Holm D.G., Suzuki D.T. (1973). Genetic control of insect populations: I. Cage studies of chromsome replacement by compound autosomes in *Drosophila melanogaster*. Genetics.

[212] James A., Handler A.M., James A.A. (2000). Control of Disease Transmission through Genetic Modification of Mosquitoes. Insect Transgenesis: Methods and Applications.

[213] Rasgon J.L., Scott T.W. (2004). *Crimson*: A Novel Sex-Linked Eye Color Mutant of *Culex pipiens* L. (Diptera: Culicidae). J. Med. Entomol..

[214] Dame D.A., Curtis C.F., Benedict M.Q., Robinson A.S., Knols B.G.J. (2009). Historical applications of induced sterilisation in field populations of mosquitoes. Malar. J..

[215] Fried M. (1971). Determination of Sterile-Insect Competitiveness. J. Econ. Entomol..

[216] Spielman A., Kitron U., Pollack R.J. (1993). Time limitation and the role of research in the worldwide attempt to eradicate malaria. J. Med. Entomol..

[217] Eckhoff P.A., Wenger E.A., Godfray H.C.J., Burt A. (2017). Impact of mosquito gene drive on malaria elimination in a computational model with explicit spatial and temporal dynamics. Proc. Natl. Acad. Sci. USA.

[218] Unckless R.L., Messer P.W., Connallon T., Clark A.G. (2015). Modeling the manipulation of natural populations by the mutagenic Chain reaction. Genetics.

[219] Unckless R.L., Clark A.G., Messer P.W. (2017). Evolution of resistance against CRISPR/Cas9 gene drive. Genetics.

[220] Robert M.A., Okamoto K.W., Gould F., Lloyd A.L. (2014). Antipathogen genes and the replacement of disease-vectoring mosquito populations: A model-based evaluation. Evol. Appl..

[221] WHO Global Programme to Eliminate Lymphatic Filariasis: Progress Report, 2014. http://www.who.int/lymphatic_filariasis/resources/who_wer9038/en/.

[222] Thomas M.B., Godfray H.C.J., Read A.F., van den Berg H., Tabashnik B.E., van Lenteren J.C., Waage J.K., Takken W. (2012). Lessons from Agriculture for the Sustainable Management of Malaria Vectors. PLoS Med..

[223] Bill and Melinda Gates Foundation From Aspiration to Action. https://www.mmv.org/sites/default/files/uploads/docs/publications/Aspiration-to-Action.pdf.

[224] Barreaux P., Barreaux A., Sternberg E., Suh E. (2017). Priorities for Broadening the Malaria Vector Control Tool Kit. Trends Parasitol..

[225] Benelli G., Jeffries C., Walker T. (2016). Biological Control of Mosquito Vectors: Past, Present, and Future. Insects.

[226] Verily. https://verily.com/projects/interventions/debug/.

[227] Eliminate Dengue Program. http://www.eliminatedengue.com/project.

[228] Shenoy R.K., Suma T.K., Kumaraswami V., Rahmah N., Dhananjayan G., Padma S. (2009). Antifilarial drugs, in the doses employed in mass drug administrations by the Global Programme to Eliminate Lymphatic Filariasis, reverse lymphatic pathology in children with *Brugia malayi* infection. Ann. Trop. Med. Parasitol..

[229] White M.T., Griffin J.T., Churcher T.S., Ferguson N.M., Basáñez M.-G., Ghani A.C. (2011). Modelling the impact of vector control interventions on *Anopheles gambiae* population dynamics. Parasit. Vectors.

[230] Fillinger U., Ndenga B., Githeko A., Lindsay S.W. (2009). Integrated malaria vector control with microbial larvicides and insecticide-treated nets in western Kenya: A controlled trial. Bull. World Health Organ..

[231] Deredec A., Burt A., Godfray H.C.J. (2008). The Population Genetics of Using Homing Endonuclease Genes in Vector and Pest Management. Genetics.

[232] Marshall J.M., Buchman A., Sanchez C.H.M., Akbari O.S. (2017). Overcoming evolved resistance to population-suppressing homing-based gene drives. Sci. Rep..

[233] Noble C., Olejarz J., Esvelt K.M., Church G.M., Nowak M.A. (2017). Evolutionary dynamics of CRISPR gene drives. Sci. Adv..

[234] Champer J., Liu J., Oh S.Y., Reeves R., Luthra A., Oakes N., Clark A.G., Messer P.W. (2017). Reducing resistance allele formation in CRISPR gene drives. bioRxiv.

[235] Esvelt K.M., Smidler A.L., Catteruccia F., Church G.M. (2014). Concerning RNA-guided gene drives for the alteration of wild populations. Elife.

[236] Gao Y., Zhao Y. (2014). Self-processing of ribozyme-flanked RNAs into guide RNAs in vitro and in vivo for CRISPR-mediated genome editing. J. Integr. Plant Biol..

[237] Port F., Bullock S.L. (2016). Augmenting CRISPR applications in *Drosophila* with tRNA-flanked sgRNAs. Nat. Methods.

[238] Yoshioka S., Fujii W., Ogawa T., Sugiura K., Naito K. (2016). Development of a mono-promoter-driven CRISPR/Cas9 system in mammalian cells. Sci. Rep..

[239] Pan X., Zhou G., Wu J., Bian G., Lu P., Raikhel A.S., Xi Z. (2012). *Wolbachia* induces reactive oxygen species (ROS)-dependent activation of the Toll pathway to control dengue virus in the mosquito *Aedes aegypti*. Proc. Natl. Acad. Sci. USA.

[240] Rancès E., Ye Y.H., Woolfit M., McGraw E.A., O’Neill S.L. (2012). The relative importance of innate immune priming in *Wolbachia*-mediated dengue interference. PLoS Pathog..

[241] Calvitti M., Marini F., Desiderio A., Puggioli A., Moretti R. (2015). *Wolbachia* density and cytoplasmic incompatibility in *Aedes albopictus*: Concerns with using artificial *Wolbachia* infection as a vector suppression tool. PLoS ONE.

[242] Akbari O.S., Antoshechkin I., Amrhein H., Williams B., Diloreto R., Sandler J., Hay B.A. (2013). The Developmental Transcriptome of the Mosquito *Aedes aegypti*, an Invasive Species and Major Arbovirus Vector. G3 (Bethesda).

[243] Macias V., Coleman J., Bonizzoni M., James A.A. (2014). piRNA pathway gene expression in the malaria vector mosquito *Anopheles stephensi*. Insect Mol. Biol..

[244] Campbell C.L., Black W.C., Hess A.M., Foy B.D. (2008). Comparative genomics of small RNA regulatory pathway components in vector mosquitoes. BMC Genom..

[245] Ronsseray S., Lehmann M., Anxolabéhère D. (1991). The Maternally Inherited Regulation of P Elements in *Drosophila* melanogaster Can Be Elicited by Two P Copies at Cytological Site 1A on the X Chromosome. Genet. Soc. Am..

[246] Kawaoka S., Mitsutake H., Kiuchi T., Kobayashi M., Yoshikawa M., Suzuki Y., Sugano S., Shimada T., Kobayashi J., Tomari Y. (2012). A role for transcription from a piRNA cluster in de novo piRNA production. RNA.

[247] Olovnikov I., Ryazansky S., Shpiz S., Lavrov S., Abramov Y., Vaury C., Jensen S., Kalmykova A. (2013). *De novo* piRNA cluster formation in the *Drosophila* germ line triggered by transgenes containing a transcribed transposon fragment. Nucleic Acids Res..

[248] Shpiz S., Ryazansky S., Olovnikov I., Abramov Y., Kalmykova A. (2014). Euchromatic transposon insertions trigger production of novel Pi- and endo-siRNAs at the target sites in the *Drosophila* germline. PLoS Genet..

[249] Le Thomas A., Stuwe E., Li S., Du J., Marinov G., Rozhkov N., Chen Y.C., Luo Y., Sachidanandam R., Toth K.F. (2014). Transgenerationally inherited piRNAs trigger piRNA biogenesis by changing the chromatin of piRNA clusters and inducing precursor processing. Genes.

[250] Boivin A., Gally C., Netter S., Anxolabéhère D., Ronsseray S. (2003). Telomeric associated sequences of *Drosophila* recruit polycomb-group proteins in vivo and can induce pairing-sensitive repression. Genetics.

[251] Cho S.W., Kim S., Kim Y., Kweon J., Kim H.S., Bae S., Kim J.-S. (2014). Analysis of off-target effects of CRISPR/Cas-derived RNA-guided endonucleases and nickases. Genome Res..

[252] Nakajima K., Kazuno A.-A., Kelsoe J., Nakanishi M., Takumi T., Kato T. (2016). Exome sequencing in the knockin mice generated using the CRISPR/Cas system. Sci. Rep..

[253] IGSR: The International Genome Sample Resource. http://www.internationalgenome.org/.

[254] Hwang W.Y., Fu Y., Reyon D., Maeder M.L., Tsai S.Q., Sander J.D., Peterson R.T., Yeh J.-R.J., Joung J.K. (2013). Efficient genome editing in zebrafish using a CRISPR-Cas system. Nat. Biotechnol..

[255] Ren X., Yang Z., Xu J., Sun J., Mao D., Hu Y., Yang S.-J., Qiao H.-H., Wang X., Hu Q. (2014). Enhanced Specificity and Efficiency of the CRISPR/Cas9 System with Optimized sgRNA Parameters in *Drosophila*. Cell Rep..

[256] Crosetto N., Mitra A., Silva M.J., Bienko M., Dojer N., Wang Q., Karaca E., Chiarle R., Skrzypczak M., Ginalski K. (2013). Nucleotide-resolution DNA double-strand break mapping by next-generation sequencing. Nat. Methods.

[257] Mali P., Aach J., Stranges P.B., Esvelt K.M., Moosburner M., Kosuri S., Yang L., Church G.M. (2013). Cas9 transcriptional activators for target specificity screening and paired nickases for cooperative genome engineering. Nat. Biotechnol..

[258] Yan W.X., Mirzazadeh R., Garnerone S., Scott D., Schneider M.W., Kallas T., Custodio J., Wernersson E., Li Y., Gao L. (2017). BLISS is a versatile and quantitative method for genome-wide profiling of DNA double-strand breaks. Nat. Commun..

[259] Hsu P., Scott D., Weinstein J., Ran F. (2013). DNA targeting specificity of RNA-guided Cas9 nucleases. Nature.

[260] Iyer V., Shen B., Zhang W., Hodgkins A., Keane T., Huang X., Skarnes W.C. (2015). Off-target mutations are rare in Cas9-modified mice. Nat. Methods.

[261] Schaefer K.A., Wu W.-H., Colgan D.F., Tsang S.H., Bassuk A.G., Mahajan V.B. (2016). Unexpected mutations after CRISPR-Cas9 editing in vivo. Nat. Methods.

[262] Zetsche B., Gootenberg J.S., Abudayyeh O.O., Slaymaker I.M., Makarova K.S., Essletzbichler P., Volz S.E., Joung J., van der Oost J., Regev A. (2015). Cpf1 Is a Single RNA-Guided Endonuclease of a Class 2 CRISPR-Cas System. Cell.

[263] Kleinstiver B.P., Pattanayak V., Prew M.S., Tsai S.Q., Nguyen N.T., Zheng Z., Joung J.K. (2016). High-fidelity CRISPR-Cas9 nucleases with no detectable genome-wide off-target effects. Nature.

[264] Slaymaker I.M., Gao L., Zetsche B., Scott D.A., Yan W.X., Zhang F. (2016). Rationally engineered Cas9 nucleases with improved specificity. Science.

[265] Ran F.A., Hsu P.D., Lin C.-Y., Gootenberg J.S., Konermann S., Trevino A.E., Scott D.A., Inoue A., Matoba S., Zhang Y. (2013). Double nicking by RNA-guided CRISPR Cas9 for enhanced genome editing specificity. Cell.

[266] Fu Y., Sandler J.D., Reyon D., Cascio V.M., Joung J.K. (2014). Improving CRISPR-Cas nuclease specificity using truncated guide RNAs. Nat. Biotechnol..

[267] Pattanayak V., Lin S., Guilinger J.P., Ma E., Doudna J.A., Liu D.R. (2013). High-throughput profiling of off-target DNA cleavage reveals RNA-programmed Cas9 nuclease specificity. Nat. Biotechnol..

[268] Haeussler M., Schönig K., Eckert H., Eschstruth A., Mianné J., Renaud J.-B., Schneider-Maunoury S., Shkumatava A., Teboul L., Kent J. (2016). Evaluation of off-target and on-target scoring algorithms and integration into the guide RNA selection tool CRISPOR. Genome Biol..

[269] Oliveros J.C., Franch M., Tabas-Madrid D., San-León D., Montoliu L., Cubas P., Pazos F. (2016). Breaking-Cas—interactive design of guide RNAs for CRISPR-Cas experiments for ENSEMBL genomes. Nucleic Acids Res..

[270] Marshall J.M. (2009). The effect of gene drive on containment of transgenic mosquitoes. J. Theor. Biol..

[271] Akbari O.S., Bellen H.J., Bier E., Bullock S.L., Burt A., Church G.M., Cook K.R., Duchek P., Edwards O.R., Esvelt K.M. (2015). Safeguarding gene drive experiments in the laboratory. Science.

[272] DiCarlo J.E., Chavez A., Dietz S.L., Esvelt K.M., Church G.M. (2015). Safeguarding CRISPR-Cas9 gene drives in yeast. Nat. Biotechnol..

[273] Oye K.A., Esvelt K., Appleton E., Catteruccia F., Church G., Kuiken T., Lightfoot S.B.-Y., McNamara J., Smidler A., Collins J.P. (2014). Regulating gene drives. Science.

[274] Wu B., Luo L., Gao X.J. (2016). Cas9-triggered chain ablation of cas9 as a gene drive brake. Nat. Biotechnol..

[275] Noble C., Min J., Olejarz J., Buchthal J., Chavez A., Smidler A.L., DeBenedictis E.A., Church G.M., Nowak M.A., Esvelt K.M. (2016). Daisy-chain gene drives for the alteration of local populations. bioRxiv.

[276] Mukundarajan H., Hol F.J.H., Castillo E.A., Newby C., Prakash M. (2017). Using Mobile Phones as Acoustic Sensors for High-Throughput Surveillance of Mosquito Ecology. bioRxiv.

[277] Grubaugh N.D., Sharma S., Krajacich B.J., Fakoli L.S., Bolay F.K., Diclaro J.W., Johnson W.E., Ebel G.D., Foy B.D., Brackney D.E. (2015). Xenosurveillance: A Novel Mosquito-Based Approach for Examining the Human-Pathogen Landscape. PLoS Negl. Trop. Dis..

[278] Oxitec. http://www.oxitec.com/.

[279] Munhenga G., Brooke B.D., Spillings B., Essop L., Hunt R.H., Midzi S., Govender D., Braack L., Koekemoer L.L. (2014). Field study site selection, species abundance and monthly distribution of anopheline mosquitoes in northern Kruger National Park, South Africa. Malar. J..

[280] Ageep T.B., Damiens D., Alsharif B., Ahmed A., Salih E.H., Ahmed F.T., Diabaté A., Lees R.S., Gilles J.R., El Sayed B.B. (2014). Participation of irradiated *Anopheles arabiensis* males in swarms following field release in Sudan. Malar. J..

[281] Lees R.S., Gilles J.R., Hendrichs J., Vreysen M.J., Bourtzis K., KBourtzis K. (2015). Back to the future: The sterile insect technique against mosquito disease vectors. Curr. Opin. Insect Sci..

[282] VectorBase: Population Biology. https://www.vectorbase.org/popbio.

[283] Pigott D.M., Kraemer M.U. Enhancing Infectious Disease Mapping with Open Access Resources. http://www.eurosurveillance.org/images/dynamic/EE/V19N49/art20989.pdf.

[284] Malaria Atlas Project. http://www.map.ox.ac.uk/explorer/.

